# Synergy Effect of Synthetic Wax and Tall Oil Amidopolyamines for Slowing Down the Aging Process of Bitumen

**DOI:** 10.3390/ma18174135

**Published:** 2025-09-03

**Authors:** Mateusz M. Iwański, Szymon Malinowski, Krzysztof Maciejewski, Grzegorz Mazurek

**Affiliations:** 1 Department of Construction Engineering, Faculty of Civil Engineering and Architecture, Kielce University of Technology, Al. Tysiąclecia Państwa Polskiego 7, 25-314 Kielce, Poland; 2 Department of Construction Materials Engineering and Geoengineering, Faculty of Civil Engineering and Architecture, Lublin University of Technology, Ul. Nadbystrzycka 40, 20-618 Lublin, Poland; s.malinowski@pollub.pl; 3 Department of Transportation Engineering, Faculty of Civil Engineering and Architecture, Kielce University of Technology, Al. Tysiąclecia Państwa Polskiego 7, 25-314 Kielce, Poland; kmaciejewski@tu.kielce.pl (K.M.); gmazurek@tu.kielce.pl (G.M.)

**Keywords:** synthetic wax, tall oil amidopolyamines, bitumen aging, chemical analysis, rheological properties, anti-aging effect

## Abstract

Bitumen ages during production and in asphalt pavements, leading to structural issues and reduced durability of asphalt pavements. The alteration of bitumen’s viscoelastic properties, predominantly attributable to oxidation phenomena, is a hallmark of these processes. This study analyzed the use of a new generation of synthetic wax (SW_LC_), which was selected for its low carbon footprint, ability to reduce binder viscosity, and ability to enable the production of WMA. Tall oil amidopolyamines (TOAs), a renewable raw material-based adhesive and aging inhibitor, was also used in this study. It compensates for the unfavorable effect of stiffening the binder with synthetic wax. SW_LC_ at concentrations of 1.0%, 1.5%, 2.0%, and 2.5% by mass in bitumen, in conjunction with TOAs at concentrations of 0.0%, 0.2%, 0.4%, and 0.6% by bitumen weight were tested at various concentrations. Short-term and long-term aging effects on penetration, softening point, and viscosity multiple creep and stress recovery tests (MSCR), oscillatory tests for the combined complex modulus |G*| and phase shift angle sin(δ) (DSR), and low-temperature characteristics S_m_ and m_value_ (BBR) were analyzed. The chemical composition of the binders was then subjected to Fourier Infrared Spectroscopy (FTIR) analysis, which enabled the determination of carbonyl, sulfoxide, and aromaticity indexes. These results indicated that the additives used inhibit the oxidation and aromatization reactions of the bitumen components. The optimal SW_LC_ and TOA content determined was 1.5% and 0.4% *w*/*w*, respectively. These additives reduce aging and positively affect rheological parameters.

## 1. Introduction

Bitumen plays a key role in ensuring the required quality of the asphalt mix and guaranteeing the long exploitation period of asphalt pavements. Therefore, it should have the most favorable physical and mechanical properties possible. To ensure high-quality bitumen, research is being conducted to obtain a binder not only with the appropriate parameters required by technical standards but also with excellent rheological parameters, ensuring the required durability of the asphalt pavement over a long period of use, while taking into account the environmental aspects of its operation.

Consequently, asphalt mixtures have been innovated to be produced and applied at lower processing temperatures, referred to as Warm Mix Asphalt or Half-Warm Mix Asphalt, as opposed to the conventional Hot Mix Asphalt. The reduction in technological temperatures of bitumen reduces “greenhouse gas” emissions, especially CO_2_, by approximately 30%, nitrogen oxides by 60% to 70%, and particulate matter by 25% to 55%, and reduces energy consumption during production by approximately 20–35% [1,2,3,4,5] thus having a significant impact on mitigating climate change on Earth. It should be noted, as demonstrated in the studies carried out, that it is difficult to clearly determine the effect in terms of the amount of “greenhouse gas” reduction, reduction in dust generated during the production process, or energy reduction. It depends on many factors, such as the type of bitumen used, the type of WMA additives, the type of asphalt mixture produced, the type of asphalt mixture plant, and climate zones. Therefore, each study focuses on finding material solutions that should ensure the most favorable environmental effects.

In order to reduce the technological temperatures of asphalt mixtures and thus of bitumen, modifying additives are used, such as synthetic wax [6,7,8], natural and waste plastics [9,10,11], derivatives of natural oils [12,13,14], surfactants [15], and various other types of chemical compounds [16,17,18]. It should be noted that the impact of these modifiers on the properties of the asphalt binder varies. They improve some characteristics and sometimes have a negative effect on other properties. A classic example is the F-T synthetic wax, which reduces the viscosity of the asphalt mixture in the range of technological temperatures of the asphalt mixture, improving the process of coating the mineral aggregates at a lower temperature than in the case of using traditional asphalt mixtures. In terms of above-zero operating temperatures, the asphalt mixture with F-T wax employed benefits from improved resistance to permanent deformation as a result of the formation of synthetic wax crystals in the bitumen. Unfortunately, in the range of sub-zero temperatures, this additive may cause a decrease in the performance of the mix, precisely as a result of synthetic wax crystals in the binder [19]. On the other hand, surface-active agents or chemical compounds added to the asphalt reduce the viscosity of the asphalt, improving the process of coating aggregate grains in the asphalt mixture, but they may adversely affect penetration and the softening point as a mark of their plasticizing effects. At the same time, they exhibit beneficial results in the sub-zero range of temperatures [20].

During the transport of bitumen from the refinery to the asphalt mixing plant, as well as its storage and subsequent production of the asphalt mixture, the bitumen is affected by high temperatures, typically up to 180 °C depending on the type of mineral and asphalt mixture produced. Then, during the service of the pavement, solar radiation, oxygen contained in the air, and climatic factors affect the bitumen further. These factors affecting bitumen cause adverse changes in its properties [21]. Since bitumen is an organic material, its processes are referred to as aging, which is defined as the occurrence of physical and/or chemical changes leading to a reduction in its performance [22]. Changes in its properties at the stage of asphalt mixture production are referred to as technological or short-term aging. On the other hand, the processes occurring during pavement service are marked as service or long-term aging. In general, the physical and chemical changes associated with bitumen aging are primarily related to the oxidation reactions of its components [22] and volatilization of low-molecular-weight fractions [23]. As a result of these asphalt processes, the stiffness and brittleness of the bitumen increase, and its adhesion to the aggregate is reduced. Thus, the asphalt mixture deteriorates in the pavement structure, which results in cracks, primarily of the wearing course, which are the result of oxidation of bitumen components [23,24,25,26] and loss of its resistance to moisture and frost. As a consequence, the quality of the asphalt surface deteriorates, and its service life is reduced, which results in a shortening of periods between rehabilitation. However, currently, for ecological, social, and logistical reasons, efforts are made to extend the exploitation period of asphalt pavements. In order to achieve this effect, research is carried out on the use of various types of agents [27,28], the task of which, as mentioned earlier, is not only to improve the physico-mechanical properties of the binder at the stage of its application to the asphalt mixture but also to provide bitumen and mixtures with greater durability [29]. Therefore, there is a new trend in the field of bitumen and asphalt pavement technology regarding the possibility of reducing the susceptibility of bitumen to aging and thus ensuring a longer exploitation period of road pavements. This trend is being implemented by developing modern additives or modifiers for bitumen. This is currently achieved through the use of a range of organic and inorganic chemical compounds, i.e., aging inhibitors [30,31]. Additionally, mineral-based materials [32], biochar [33], antioxidants [34,35], and biomaterials [32,36] were used to this end, including oils of organic origin, e.g., fatty acid methyl ester-based additive from sunflower oil [37], polyaniline nanofibers [21], or cellulose [38]. It should be noted that, among the organic compounds, the anti-aging activity of SBS copolymers has been found [34,39,40,41], which is often used as a bitumen modifier to improve its standard and rheological properties. Unfortunately, for technological reasons, it is not used in WMA technology. Some work was also carried out to identify the possibility of using selected additives and modifiers used to improve physico-mechanical and rheological properties of bitumens to act as agents slowing down or limiting the aging process of the binder.

The investigations usually concerned the use of only one additive/modifier to the bitumen. On the other hand, interesting results were obtained by conducting preliminary studies on the use of two additives/modifiers, i.e., SW_LC_ synthetic wax and a surfactant obtained on the basis of TOAs (tall oil amidopolyamines). Their impact on bitumen complemented each other, compensating for the adverse effects of one additive by the other and enhancing their beneficial effect [20,42,43]. Therefore, the research presents the development of the first work on the use of both additives in the modification and increase in their effect on the anti-aging resistance of road bitumen. The effect of both additives on the process of technological (short-term) and operational (long-term) aging and chemical and rheological properties was studied.

This study focuses on extending pavement service life and enabling low-temperature asphalt production. By reducing material aging and associated maintenance cycles, our research promotes sustainable transportation infrastructure and minimizes the environmental footprint of road networks. This aligns with global efforts to improve the eco-efficiency of transport systems and achieve long-term sustainability goals, as evidenced by the literature on green pavement technologies [44], road pavement rehabilitation [45,46], and the broader imperative for environmentally responsible transport development [47].

The work was based on the following hypotheses:

1. The addition of low-carbon footprint synthetic wax (SW_LC_) and tall oil amidopolyamines (TOAs) reduces the aging rate of bitumen.
2. The synergy of SW_LC_ and TOAs has a positive effect on the chemical and rheological properties of the binder in the aging process.

**Scheme 1:**
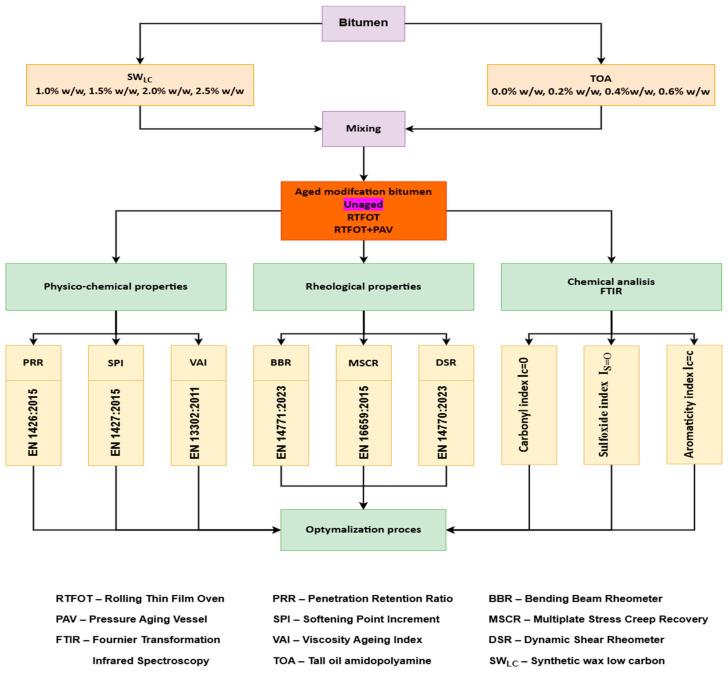
General testing plan.

In order to verify the mentioned hypotheses, physical and mechanical properties of bitumens were tested, taking into account the aging process. The aging characteristics of asphalt binders were determined, and the effect of aging on the chemical and rheological properties of bitumen modified with SW_LC_ and TOA was examined. In order to verify the hypotheses, the necessary tests and their analyses presented in Scheme 1 were performed.

These included analysis of the effects of SW_LC_ and TOA on the chemical, physical, and rheological properties of bitumen. The process of limiting physicochemical changes in bitumen as a result of aging was evaluated using the PRR (penetration retention ratio), SPI (softening temperature increment), and VAI (viscosity aging index). On the other hand, in order to identify the phenomenon of chemical changes, Fourier transform infrared spectroscopy (FTIR) tests were performed. It should be noted that all asphalt compositions with the addition of SW_LC_ and TOA were tested before and after technological (RTFOT) and operational (RTFOT + PAV) aging, and then the carbonyl index (I_C=O_), sulfoxide index (I_S=O_), and aromaticity index (I_C=C_) were determined. The determined indices directly reflect the amount of carbonyl and sulfoxide groups formed by oxidation reactions and benzene rings formed by aromatization. Consequently, there is a change in the physical and mechanical properties of the binder composition (bitumen + synthetic wax + tall oil amidopolyamine), which has a significant impact on the behavior of asphalt mixtures in the layers of the road pavement structure and affects its durability.

## 2. Materials and Methods

### 2.1. Materials

This study used 50/70 bitumen from Orlen Asfalt SA in Płock, Poland, which is the basic bitumen used for asphalt mixtures intended for the upper layers of the pavement structure in the countries of Central and Eastern Europe. The basic properties of the base bitumen are listed in Table 1. On the other hand, WMA additives were used as modifiers of bitumen properties. SW_LC_ and TOA were used. As a result of the synergy of their interaction with bitumen, their task is to regulate the aging of bitumen and to counteract adverse changes in its physical and mechanical properties.

### 2.2. Synthetic Wax LC and Tall Oil Amidopolyamines

Two types of WMA additives to bitumen were used in this study.

As one of the additives for the 50/70 bitumen, Sasobit LC (SASOL Chemicals, Hamburg, Germany), a new generation of F-T synthetic wax (SW_LC_), was used, characterized by a reduced carbon footprint compared to the one used so far in road practice. It was introduced into road practice in 2023 as a material that meets the current high environmental requirements. The low carbon footprint of SW_LC_ is confirmed by the manufacturer’s data from an LCA analysis, taking into account emissions during production and transport, which are included in the product specifications [52].

**Figure 1:**
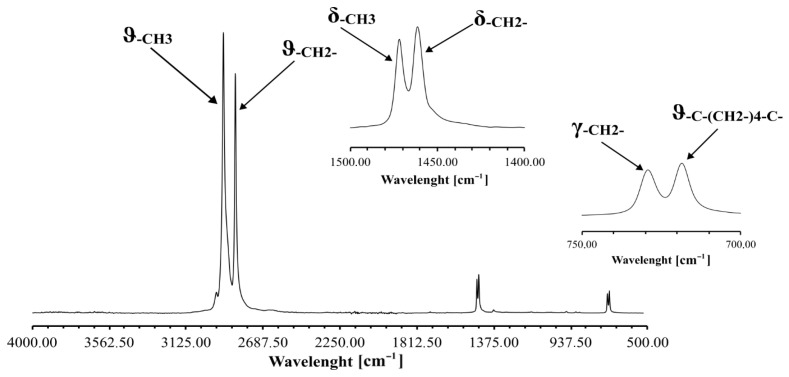
FTIR spectra of SW_LC_.

Synthetic F-T wax differs significantly from the natural paraffin found in petroleum. It is a long-chain hydrocarbon (its hydrocarbon chains contain between 40 and 115 carbon atoms) obtained from CO and H_2_ in the Fischer–Tropsch synthesis process. It has a more finely crystalline structure than natural paraffin, whose hydrocarbon chains contain between 15 and 50 carbon atoms. Its molecular weight is about 40% higher than that of the natural waxes found in bitumen. The long hydrocarbon chains of synthetic F-T wax crystallize at higher temperatures, and the shorter chains crystallize at lower temperatures. Their effect is similar to that of asphaltenes and depends on the reduction in their mobility with respect to the maltene fraction [11]. The presence of hydrocarbons in the chemical structure of SW_LC_ is confirmed by the FTIR spectrum shown in Figure 1. It shows 3 doublets of characteristic bands: (I) at 2900 cm^−1^ and 2800 cm^−1^ corresponding to stretching vibrations of C-H bonds in -CH_3_ and -CH_2_- groups, (II) at 1470 cm^−1^ and 1460 cm^−1^ corresponding to deformation vibrations of -CH_3_ and -CH_2_- groups, and (III) at 730 cm^−1^ and 720 cm^−1^ corresponding to skeletal vibrations of hydrocarbon chains with a minimum of 4 carbon atoms.

**Figure 2:**
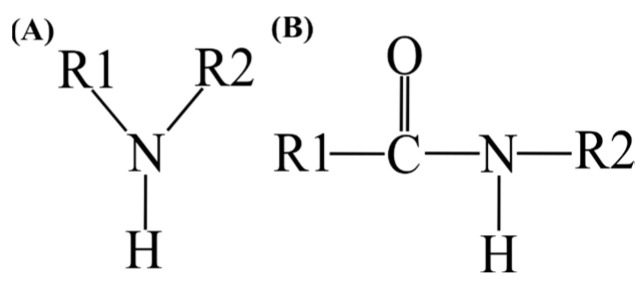
Chemical structure of amine (**A**) and amide (**B**) functional groups.

**Figure 3:**
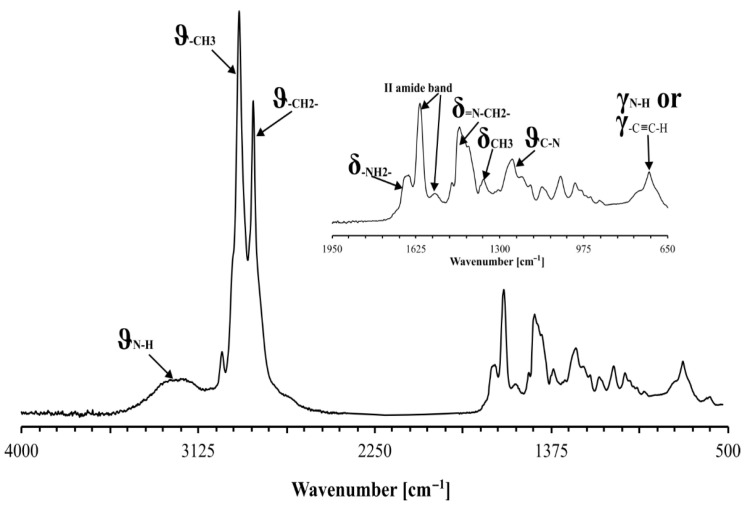
FTIR spectra of TOA.

The second additive used is tall oil amidopolyamine (TOA), manufactured by Akzo Nobel Surface Chemistry, Nouryon, Amsterdam, in the Netherlands [53]. It is insoluble in water and soluble only in certain organic solvents (e.g., toluene, paraffin, trichloroethylene). This agent lowers the surface tension of the bituminous binder, thereby improving the coating of aggregate particles with bitumen in the asphalt mixture production process. This agent acts as a surfactant (SAA). A characteristic feature of compounds from the polyamine group is the presence of at least two amine groups, shown schematically in Figure 2. Their appearance can be confirmed by the presence of bands located on the FTIR spectrum of TOA (Figure 2A) at wavelengths of 3200 cm^−1^, 1650 cm^−1^, 1460 cm^−1^, and 1220 cm^−1^ corresponding to stretching and deformation vibrations of the secondary amine group, stretching vibrations of the =N-CH_2_- group, and stretching vibrations of the C-N bond of the secondary amine group, respectively. The FTIR spectrum also indicates that TOA contains amide groups (Figure 2B) in its chemical structure. Their presence is indicated by the presence of characteristic bands at 1610 cm^−1^ and 1550 cm^−1^ attributed to the 2nd amide band of the primary and secondary amides, respectively. The presence of an amide group is also indicated by the presence of a band at 720 cm^−1^ corresponding to the fan vibration of the N-H bonds. In addition, methyl and methylene groups are also evident in the chemical structure of TOA, both on the basis of bands derived from C-H bond stretching vibrations located at 2920 cm^−1^ and 2850 cm^−1^ respectively, as well as a band located at 1370 cm^−1^ corresponding to deformation vibrations of the -CH_3_ group. The FTIR spectra of TOA is shown in Figure 3.

The basic physical properties of SW_LC_ and TOA are presented in Table 2.

### 2.3. Synthetic Wax LC and Tall Oil Amidopolyamines—Modified Bitumen Preparation

Due to the fact that bitumen additives were used in the research, special attention was paid to obtaining homogeneity of the modified binder and the samples prepared from it for testing. In total, 50/70 binders with 16 combinations of additives were tested: SW_LC_ in the range of 1.0–2.5% *w*/*w* (with increments of 0.5%) and TOA 0–0.6% *w*/*w* (with increments of 0.2%). TOAs were added to the 50/70 bitumen samples weighing 1000 g in the amounts required by the experiment. Mixing the binder with the additive consisted of heating the binder to a temperature 100 °C higher than its softening point and mixing it in a blender at this temperature using a stirrer rotating at a speed of 150 rpm for 30 s and then at 600 rpm for 270 s. The temperature at which TOA is added to the binder, approximately 150–160 °C, is lower than the thermal degradation limit of amides, which is >200 °C, and the exposure time is short, which minimizes additional oxidation of the bitumen. Analytical samples were subsequently prepared for testing in compliance with EN 12594 [54]. A macroscopic evaluation of the surface of the obtained binder samples was conducted to determine their homogeneity. In instances where a lack of uniform binder coloration was observed or various types of surface spots were identified on the analytical sample, which indicated an irregular dissolution of TOA, such samples were excluded from the tests. The SW_LC_ synthetic wax was introduced into the binder, prepared in its liquid form and then heated to the specified experimental amount. Thereafter, the binder was mixed in accordance with the previously outlined procedure.

After obtaining the SW_LC_ and TOA modified binder, its homogeneity was evaluated. First, the surface of the obtained binder samples was macroscopically evaluated, and then its morphology was assessed using an AXIOI Scope.A1 epifluorescence microscope from Carl Zeiss Microlmaging, Oberkochen of Germany. Photographs of binder samples were compared and analyzed for homogeneity using software ImageJ 1.54k [55]. Only binder samples meeting homogeneity requirements were used for this study. Tests on the properties of bitumen with SW_LC_ and TOA were performed immediately after binder preparation according to the program shown in Scheme 1.

### 2.4. Aging Procedure

The bitumen aging process was carried out in two stages. In the first stage, the RTFOT (Rolling Thin Film Oven Test) was carried out in accordance with the requirements of EN 12607-1 [56], using a device made by MATEST S.p.A., Treviolo of Italy. The aging simulation was carried out based on nine samples. Then, the binders were subjected to long-term aging using the PAV (Pressure Aging Vessel) procedure according to EN 14769 [57] in a pressure-thermal chamber model 9300 PAV System from Prentexa Alloy Fabricators, Inc. Dallas, TX, USA. The test was performed on samples weighing 50 ± 0.5 g each, stored at a temperature of 100 °C and a pressure of 2.1 MPa for 20 h. According to EN 14769 [57], these test parameters correspond to aging equivalent to approximately 7–10 years of exploitation in a temperate climate. The aging simulation was carried out based on nine samples. The modified bitumen 50/70 parameters were evaluated on binder samples prepared using this method.

### 2.5. FTIR Procedure

FTIR spectra of the investigated bitumen were recorded using Nicolet iS5 with PIKE GladiATR, Madison, WI, USA in the wavelength range 4000 cm^−1^–525 cm^−1^ at 0.482 cm^−1^ resolution and 32 scans. FTIR analysis of all bitumen samples was repeated a minimum of 5 times. These were based on recorded spectra chemical indexes, i.e., carbonyl index (I_C=O_), sulfoxide index (I_S=O_), and aromaticity index (I_C=C_), quantitatively describing the structural changes in bitumen values determined by Equations (1)–(3):

(1) $$I_{C=O}=\frac{A_{1700}}{\sum A},$$

(2) $$I_{S=O}=\frac{A_{1030}}{\sum A},$$

(3) $$I_{C=C}=\frac{A_{1600}}{\sum A},$$

where A_1700_ is the area of the peak located at a wavenumber of 1700 cm^−1^, A_1030_ is the area of the peak located at a wave number of 1030 cm^−1^, A_1600_ cm^−1^ is the area of the peak located at a wave number of 1600 cm^−1^, and ΣA is the sum of the areas of the peaks located at the following wave numbers: 2900–2800 cm^−1^, 1700 cm^−1^, 1600 cm^−1^, 1450 cm^−1^, 1380 cm^−1^, 1030 cm^−1^, and 900–700 cm^−1^. Spectroscopic analysis was carried out to evaluate the impact of the short- and long-term aging process of bitumen modified with SW_LA_ and TOA based on ΔI_C=O_ RTFOT/ΔI_C=O_ RTFOT+PAV, ΔI_S=O_ RTFOT/ΔI_S=O_ RTFOT+PAV, and ΔI_C=C_ RTFOT/ΔI_C=C_ RTFOT+PAV values determined by Equations (4)–(6):

(4) $$\Delta I_{C=O}^{RTFOT/RTFOT+PAV}=I_{C=O}^{RTFOT/RTFOT+PAV}-I_{C=O},$$

(5) $$\Delta I_{S=O}^{RTFOT/RTFOT+PAV}=I_{S=O}^{RTFOT/RTFOT+PAV}{-I}_{S=O},$$

(6) $$\Delta I_{C=C}^{RTFOT/RTFOT+PAV}=I_{C=C}^{RTFOT/RTFOT+PAV}-I_{C=C},$$

### 2.6. Methods for Bitumen Testing

The base 50/70 bitumen and the SW_LC_ and TOA modified bitumens were tested before and after RTFOT and RTFOT + PAV aging in terms of penetration, softening point, and dynamic viscosity. The tests were carried out according to the experimental plan. Bitumen penetration was determined in accordance with EN 1426:2015 [48] at 25 °C using a semi-automatic penetrometer from FrӧWag Frohlich + Wagner GmbH, Obersulm of Germany. The softening point of the tested bitumen was determined by the “ring and ball” method according to EN 1427:2015 [49] using an automatic device for measuring the softening temperature of bitumen from FrӧWag Frohlich + Wagner GmbH, Obersulm of Germany. The dynamic viscosity of bitumen was determined using a rotational viscometer at T = 135 °C according to ASTM D 4402 [58] using DHR-2 Dynamic Shear Rheometer from TA Instruments DISCOVERY HR-2 rheometer made in the New Castle, DE, USA. Based on the results obtained for the aged and unaged binder, quantitative aging indices were determined [59], i.e., PRR, SPI and VAI, which were calculated according to Equations (7)–(9):

(7) $$PRR=\frac{{Penetration}_{RTFOT\;or\;RTFOT+PAV}}{{Penetration}_{unaged}\;}\cdot 100\%,$$

*SPI* = *Softening point*_RTFOT or RTFOT+PAV_ − *Softening point_unaged_*, (8)

(9) $$VAI=\frac{{Viscosity}_{RTFOT\;or\;RTFOT+PAV-\;{Viscosity}_{unaged}}}{{Viscosity}_{unaged}}\cdot 100\%,$$

The BBR test was conducted to evaluate the low-temperature resistance of the binder. The test was performed on a BBR 2S apparatus from Applied Test Systems, Butler, PA, USA. For the binder test, bales of 125/12.5/6.25 mm were prepared from binders previously subjected to RTFOT and RTFOT + PAV aging. In order to determine the parameters of the test very accurately, it was performed at four temperatures: −10 °C, −16 °C, −22 °C, and −28 °C. During the test, the deflection of the beam was recorded continuously, and then the creep stiffness parameter (S) and the creep rate value (m) were determined according to EN 14771:2023 [60].

The Multiple Stress Creep Recovery (MSCR) test was performed in a DHR-2 Dynamic Shear Rheometer DST from TA Instruments DISCOVERY HR-2 made in the New Castle, DE, USA in accordance with EN 16659 [61]. It included cyclic creep tests with relaxation simulation of the rutting process, which corresponded to the elastic response of bitumen to shear creep and recovery. Short-term and long-term aging procedure of the asphalt was performed before the test. The MSCR test was performed by inflicting 10 one-second creep cycles at a stress of 0.1 kPa and 3.2 kPa followed by relaxation of the specimens for 9 s. The values of irreversible creep susceptibility (Jnr) and elastic recovery (R) were determined from the strain values after each stage of the cycles by testing at least three specimens.

Tests of the combined dynamic modulus |G*| and phase shift angle sin(δ) of bitumen were performed in a dynamic shear rheometer from TA Instruments DISCOVERY HR-2 made in the New Castle, DE, USA and determined at temperatures from 10 °C to 90 °C in 10 °C steps at frequencies from 0.16 to 16 Hz under controlled deformation conditions according to EN 14770:2012 [62] on at least three specimens. Based on the experimental data, leading curves of the composite shear modulus were constructed according to the Christensen Anderson Marasteanu (CAM) model [10,63]. The Christensen Anderson Marasteanu (CAM) model is a modification of the Christensen Anderson (CA) [64] model and allows the model to better match the results of dynamic modulus |G*| measurements located in the very low and high frequency ranges [63,64]. It represents an attempt to improve the description of the stiffness behavior of both ordinary and modified bitumen.

The dynamic modulus |G*| and the phase shift angle δ are determined using Equations (10) and (11) [27,65]:

(10) $$\left|G^{*}\right|=G_{g}\cdot {\left[1+{\left(\frac{f_{c}}{aT\cdot f}\right)}^{k}\right]}^{-\frac{me}{k}},$$

(11) $$\delta =\frac{90m_{e}}{\left[1+{\left(\frac{f_{c}}{aT\cdot f}\right)}^{k}\right]},$$

where $\left|G^{*}\right|$ —dynamic modulus, $G_{g}$ —glassy modulus, $f_{c}$ —crossover frequency, $a_{T}$ —horizontal shift factor, f —frequency, k and m_e_—curve fitting parameters, and δ—phase shift angle.

The CAM model defines the rheological index Rc are determined using Equation (12):

(12) $$Rc=\frac{log2}{k},$$

The horizontal shift factor was defined using the William–Landel–Ferry relationship and determined using Equation (13):

(13) $$loga_{T}=\frac{C_{1}(T-T_{0})}{C_{2}+(T-T_{0})},$$

where $C_{1},\;C_{2}$ —constants, T —testing temperature, and $T_{0}$ —reference temperature (50 °C, set as a midpoint of the testing range).

### 2.7. Design of Experiment

The experiment design was based on the assumptions of the factorial algorithm of the experiment design [65]. The properties of the modified 50/70 bitumen with the addition of SW_LC_ and TOA were determined on the basis of the assumed 4 × 4 factorial design, in accordance with the adopted test program shown in Scheme 1.

The choice of model class depended on the degree to which it explained the variability of the experimental data and was based on analysis of variance. In addition, a “lack of fit” analysis was performed to indicate whether the explanation of the variability provided by the model is greater than the spread of the random estimation error.

In the next stage, the coefficients of the polynomial with degree were estimated on the basis of the ANOVA analysis of variance. The approximation of the parameters was based on the least squares method [66].

Therefore, in order to comprehensively describe the change in the value of the tested parameter (y) in bitumen 50/70 in relation to the content of SW_LC_ and TOA, a statistical model using a polynomial of the second degree was adopted [66,67] and determined using Equation (14):

(14) $$y=b_{0}+\sum_{i=1}^{n}b_{i}\cdot x_{i}+\sum_{i=j=1}^{n}b_{i=j}\cdot x_{i}\cdot x_{j}+\sum_{i=1}^{n}b_{ii}\cdot x_{ij}^{2},$$

where x_i_ = synthetic wax—SW_LC_ (%), x_j_ = tall oil amidopolyamine—TOA (%), and b_0_–b_5_—regression coefficients

A modified coefficient of determination, R^2^, was used as an indicator of the quality of explanation of the variability of the experimental data. The results of this research were visualized using the Statistica software 13.3 [68].

The number of samples used in the tests on which the determinations were made depended on the requirements of the standards in question but could not be lower than the number of estimated parameters of the model (14). Therefore, the sample quantity ranged from 5 to 9 for each combination of SW_LC_ and TOA included in the experimental design as recommended [66].

In order to determine the optimal amount of SW_LC_ and TOA in bitumen, multi-criteria statistical optimization with a generalized desired value function was used [67,69,70]. The range of acceptable parameter values for a given material was determined by the lower and upper limits of the requirements set for them. Generally, values of “0” are considered unacceptable, and “1” characterizes the most desirable values of a given feature [69,70].

Evaluating one or two material characteristics using the same values may be easy, but when more parameters and alternatives are available, it is considered advisable to combine these separate values into a desirability index (D), which allows for easier comparisons. The desirability index was calculated as the geometric mean D of the approval values according to Equation (15):

(15) $$D={\left[\prod_{u=1}^{n}d_{u}\right]}^{\frac{1}{n}},$$

where

n—number of variables, d_u_—values of individual desirability.

The optimal contents of the analyzed material components (SW_LC_ and TOA) determined using the desirability function are presented graphically using Statistica software 13.3 [68].

## 3. Results and Discussion

### 3.1. The Effect of the SW_LC_ Synthetic Wax and TOA Amount on the Bitumen Physico-Mechanical Properties

**Figure 4:**
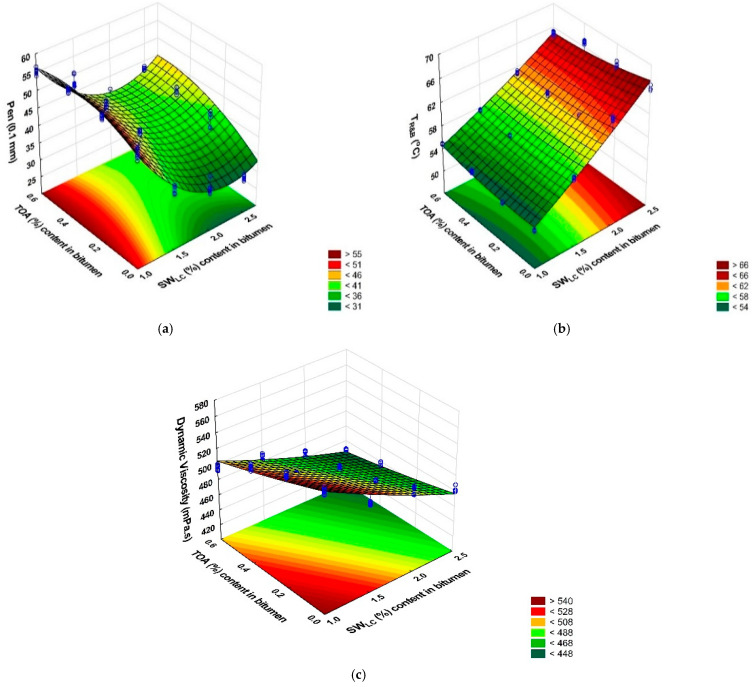
Penetration (**a**), softening point (**b**), and dynamic viscosity (**c**) of the bitumen modified with SW_LC_ and TOA.

During the research, the main physico-mechanical properties of the modified 50/70 bitumen, which include penetrations at 25 °C (Pen_25_), softening point (T_R&B_), and dynamic viscosity (η_135_), were determined, according to Scheme 1. Tests were performed on nine samples in a given series, established on the basis of subject standards and own research [66]. A comprehensive analysis of the change in Pen_25_, T_R&B_, and η_135_ of bitumen 50/70 in terms of the amount of W_LC_ and TOA is presented using regression models using the relation (14) in the form of response surfaces generated using the Statistica software [68] (Figure 4).

The significance of the influence of each binder component and the interaction between components on the change in Pen25 (0.1 mm), T_R&B_ (°C), and η135 (Pa·s) was determined using ANOVA [68], as shown in Table 3.

The analysis of statistical parameters in Table 3 clearly shows that the SW_LC_ and TOA content had a significant effect on Pen25 (0.1 mm), T_R&B_ (°C), and η135 (Pa·s) of the adhesive, as the associated *p*-value is less than the selected significance level α = 0.05 (*p*-value < 0.05). It should also be noted that there is a synergistic effect of SW_LC_ and TOA on the analyzed characteristics of the binder.

On the basis of the results of this research, it can be concluded that, with the increase in the amount of SW_LC_ in the bitumen, the penetration decreases, which is an adverse effect of its stiffening role in the binder, and corresponds to the research [70] (Figure 4a). However, in the case of the use of the TOA, no such tendency is observed. In the TOA dosing range of more than 0.2% of the above-mentioned bitumen 50/70, its beneficial effect on penetration is observed in the entire SW_LC_ dosing range. The most beneficial synergy effect of dosing of both additives on binder penetration is observed in the dosage range of SW_LC_ exceeding 1.5% and TOA exceeding 0.2%.

Synthetic wax added in amounts larger than 1.5% and TOA significantly affect the softening point of the modified binder (Figure 4b). There was no significant increase in the softening point of the modified binder in the range of up to 1.5% SW_LC_ or in the entire TOA dosing range. The softening point of the modified bitumen at this level of additive dosing does not exceed 50 °C. On the other hand, an increase in the amount of synthetic wax above 1.5% causes a significant increase in the softening point, regardless of the amount of dosed TOA, which reaches the maximum value with a synthetic wax content of 2.5% at 68 °C. It should be noted that a beneficial effect of TOA is observed, causing a decrease in the softening point as its concentration in the bitumen increases.

The use of SW_LC_ significantly reduces the dynamic viscosity of the modified 50/70 bitumen (Figure 4c). It is particularly significant with its concentration of 2.0% in the binder. With the content of TOA above 0.2%, a synergy effect of the effect of both additives is observed, reducing the dynamic viscosity, which reaches the value in the range of 400 mPa·s. Of course, this phenomenon is beneficial because it allows the aggregate grains of the asphalt mixture to be properly coated at a lower temperature than in the traditional production process. On the other hand, for modified bitumen with a content of SW_LC_ and TOA, respectively, up to 1.5% and 0.2%, dynamic viscosity remains in the range of up to 520 mPa·s.

In conclusion, it can be stated that a synergy effect of the effect of SW_LC_ and TOA on penetration at 20 °C, softening point, and dynamic viscosity of modified bitumen is observed. The intensity of the effect of both additives on the analyzed properties of the binder varies and depends on their concentration. Synthetic wax causes stiffening of the binder, and TOA causes its reduction. By using both additives and the synergy occurring between them, depending on their concentration, the parameters of the modified binder can be adjusted, taking into account its purpose in terms of the type of asphalt mixture. This was confirmed by the results of the analysis presented in Table 3. However, it is generally observed that the use of SW_LC_ in the amount of 1.5% and TOA in the amount of 0.4% provides its beneficial properties.

### 3.2. The Effect of the SW_LC_ and TOA on Long- and Short-Term Bitumen Aging

**Figure 5:**
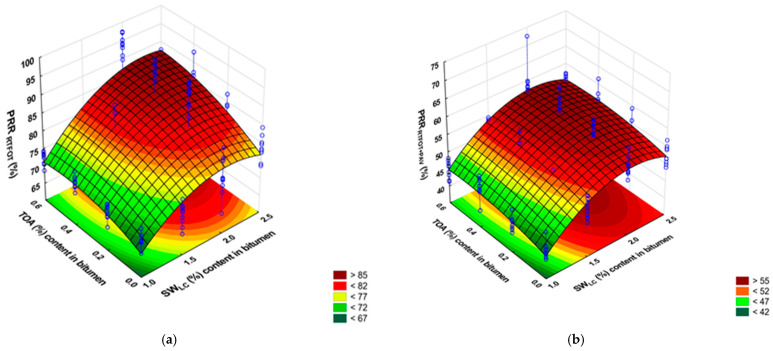
Penetration retention ratio after RTFOT (**a**) and RTFOT + PAV (**b**) of the bitumen modified with SW_LC_ and TOA.

The effect of short-term (RTFOT) and long-term (RTFOT + PAV) aging on the changes in penetration at 25 °C of bitumen 50/70 in terms of the amount of SW_LC_ and TOA was characterized by the aging indices’ penetration retention ratios (PRR_RTFO_ and PRR_RTFOT+PAV_) and are presented using regression models using the relationship (14) in the form of response surfaces generated using the Statistica software 13.3 (Figure 5).

The analysis of the significance of each binder component and the interaction between components for the variability of PRR_RTFO_ and PRR_RTFOT+PAV_ was performed using analysis of variance, ANOVA [68] (Table 4).

The analysis of statistical parameters in Table 4 clearly shows that the SW_LC_ and TOA content had a significant impact on the aging characteristics of the PRR_RTFO_ and PRR_RTFOT+PAV_ binders, as the *p*-value associated with it is less than the accepted significance level of α = 0.05 (*p*-value < 0.05).

An analysis of penetration at 25 °C after the RTFOT process of bitumen 50/70 (Figure 5a) based on the PRR_RTFOT_ aging index showed a positive synergy effect of SW_LC_ and TOA on the change in this parameter. Dosing of SW_LC_ in the range of up to 1.5% and TOA in the amount from 0.0% to 0.6% of bitumen provides the smallest reduction in penetration amounting to up to 42% change in its initial value. Therefore, the bitumen will not undergo excessive stiffening as a result of aging, which will ensure its proper operation in the asphalt mixture over the long life pavement. On the other hand, increasing the amount of SW_LC_ above 1.5% with TOA in the entire range of its dosage results in a significant reduction in penetration at 25 °C after the RTFOT aging process, reaching up to 58% of the change in its value before aging. The -RTFOT + PAV process (Figure 5b) causes further unfavorable reduction in the penetration at 25 °C of asphalt with the addition of SW_LC_ and TOA. The bitumen is further hardened. It should be noted that, with the content of TOA in the range from 0.0% to 0.6% and SW_LC_ in the amount of up to 1.5%, the penetration value increases by as much as 70%.

**Figure 6:**
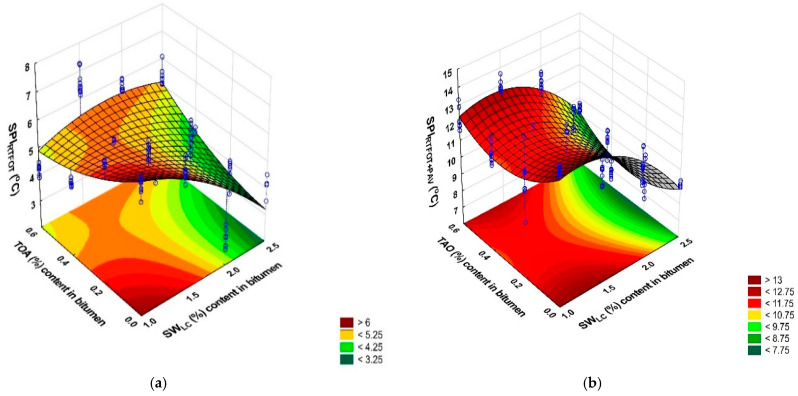
Softening point increment: RTFOT (**a**) and RFTOT + PAV (**b**) of the bitumen modified with SW_LC_ and TOA.

The effect of RTFOT short-term and RTFOT + PAV long-term aging on softening point in terms of the amount of the additives was presented using regression models using the relationship (14) in the form of response surfaces generated using the Statistica software 13.3 [68]. Regression models of the SPI_RTFOT_ and SPI_RTFOT+PAV_ softening point aging indices are presented in Figure 6. Tests were performed on nine samples in each series of binder composition [66].

The influence of the significance of each binder component and the interaction between components on the variability of SPI_RTFOT_ and SPI_RTFOT+PAV_ was performed using ANOVA analysis [68] (Table 5).

The analysis of the statistical parameters presented in Table 5 confirms the conclusion that the SW_LC_ and TOA content is a significant factor influencing the characteristics of SPI_RTFOT_ and SPI_RTFOT+PAV_. The *p*-value is less than the selected significance level α = 0.05 (*p*-value < 0.05). There is also a synergistic effect of SW_LC_ and TOA on the analyzed characteristics of the binder.

Assessing the relationships presented in Figure 6a, it can be concluded that, in the case of the use of SW_LC_ in the amount from 2.0% to 2.5% and TOA in the range from 0.0% to 0.4% of bitumen, a positive synergy effect is observed. The softening point of asphalt after the short-term aging process increased only by about 4.0 °C. On the other hand, the softening point of asphalt 50/70 increased by about 5.0 °C. On the other hand, its largest increase occurs in the scope of SW_LC_ dosing in the amount from 1.0% to 1.5% of the above-mentioned bitumen and the amount of TOA from 0.0% to 0.2% of the above-mentioned bitumen, and it amounts to the range of 5.0 °C to 6.0 °C. Therefore, the modified bitumen will have a positive effect on the structure of the asphalt mixture, ensuring the required durability of the asphalt surface.

On the basis of the analysis of the data presented in Figure 6b, it can be concluded that, in the long-term aging process, SW_LC_ in the amount of 2.0% to 2.5% and TOA in the dosing range from 0.0% to 0.6% play a similar role as in the short-term aging process. In this range of asphalt additive dosing, the softening point after long-term aging increases in the range of 7.0 °C to 10.0 °C, and the softening point of binder 50/70 increased by about 15 °C. However, in the remaining range of additive dosing, the increase in softening point reaches up to 13 °C.

**Figure 7:**
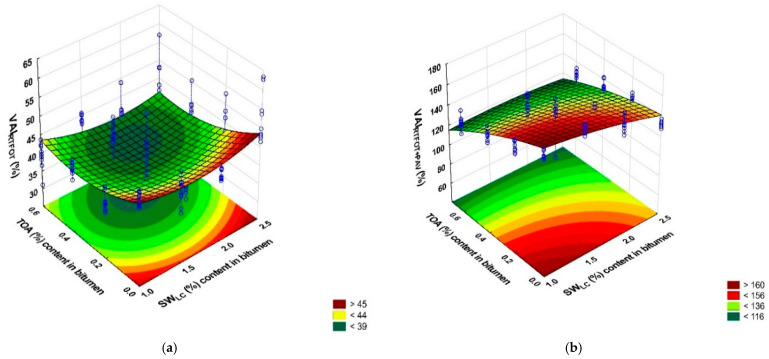
Viscosity aging index: RTFOT (**a**) and RTFOT + PAV (**b**) of the bitumen modified with SW_LC_ and TOA.

Regression models showing the effect of RTFOT and RTFOT + PAV aging and aging on changes in the dynamic viscosity of bitumen 50/70 in terms of the amount of SW_LC_ and TOA were presented using the relation (14) in the form of response surfaces generated using the ANOVA [68] (Figure 7).

The influence of the significance of each binder component and the interaction between components on the variability of VAI_RTFOT_ and VAI_RTFOT+PAV_ was performed using analysis of variance, ANOVA [68] (Table 6).

The analysis of statistical parameters in Table 6 clearly shows that the SW_LC_ and TOA content had a significant impact on the characteristics of VAI_RTFOT_. In the case of VAI_RTFOT+PAV_ binders, TOA plays a significant role. It should be noted that, in the case of the VAI_RTFOT+PAV_ analysis, a synergistic effect of the WMA additives used in the tests is observed, as the *p*-value associated with it is lower than the selected significance level α = 0.05 (*p*-value < 0.05).

By analyzing the relationships presented in Figure 7a in terms of the effect of additives after the short-term aging process on the dynamic viscosity, it can be stated that TOAs have a beneficial effect in the dosing range from 0.2% to 0.6% bitumen. This effect is even more favorable with simultaneous application of SW_LC_ in the amount of 1.5% regarding asphalt binder mass. A synergy of the effects of both additives is observed, which has a positive effect on the dynamic viscosity of bitumen after RTFOT aging. On the other hand, Figure 4b indicates that the dynamic viscosity increase in RTFOT + PAV aged bitumens with additives is the most inhibited with TOA in amounts from 0.2% to 0.6% regardless of the SW_LC_ dosing. Therefore, by using specific amounts of additives, it is possible to ensure the correct dynamic viscosity of the bitumen, taking into account aging, thus affecting the fatigue life of the asphalt mix.

### 3.3. FTIR Analysis of SW_LC_ and TOA Modified Bitumen Before and After Aging Process

**Figure 8:**
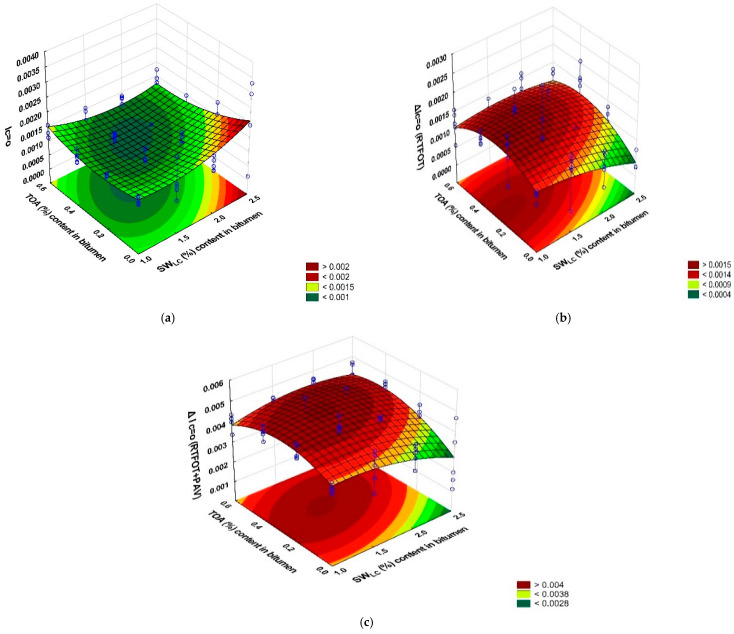
I_C=O_ indexes of bitumen modified with SW_LC_ and TOA before (**a**) and ΔI_C=O_ change after RTFOT (**b**) and RTFOT + PAV (**c**).

Firstly investigation was conducted into how modification of 50/70 road bitumen with SW_LC_ and TOA affects the I_C=O_ value, directly describing the amount of polar C=O groups before and after RTFOT and RTFOT + PAV, using the regression models developed in accordance with (14), and the response surface is shown in Figure 8. The significance analysis of each binder component and the interaction between components for the variability I_C=O_, ΔI_C=O_ RTFOT, and ΔI_C=O_ RTFOT + PAV was performed using ANOVA analysis [68] (Table 7).

The analysis of statistical parameters in Table 7 clearly shows that the SW_LC_ and TOA content had a significant impact on I_C=O_, ΔI_C=O_ RTFOT, and ΔI_C=O_ RTFOT + PAV, as the associated *p*-value is lower than the selected significance level α = 0.05 (*p*-value < 0.05).

Figure 5a shows that the introduction of 1.0%, 1.5%, 2.0%, and 2.5% SW_LC_ into the bitumen matrix increased I_C=O_. In comparison to the reference bitumen, the observed increases were 64%, 52%, 49%, and 209%, respectively. This increase may be due to the oxidation of saturated hydrocarbons with the sum formula C_n_H_2n+2_ occurring during the bitumen modification process. The modification process is carried out at a high temperature, which may facilitate the oxidation of hydrocarbons with low C atoms. However, such remarkable changes were not observed for 50/70 road bitumen modified with TOA alone. Figure 8a shows that its application in amounts of 0.2% and 0.6% caused an increase in the I_C=O_ of 4% and 25%, respectively. On the other hand, the addition of TOA at 0.4% to the bitumen matrix resulted in a reduction in I_C=O_ of about 12% compared to neat bitumen. However, simultaneous application of SW_LC_ at 1.0% and TOA at 0.2%, 0.4%, and 0.6% showed similar I_C=O_ rises ranging from 49% to 54%. Slightly greater I_C=O_ changes were observed for samples containing 1.5% SW_LC_ and various amounts of TOA. Their ratio of 1.5:0.2, 1.5:0.4, and 1.5:0.6 showed an increase in I_C=O_ of about 45%, 60%, and 51%, respectively. A completely different trend was observed for bitumen containing 2.0% SW_LC_ and varying amounts of TOA. In this case, an increase in I_C=O_ values of about 58% and 45% was observed for bitumen containing modifiers in ratios of 2.0:0.2 and 2.0:0.6, respectively. However, for bitumen containing SW_LC_ and TOA in a ratio of 2.0:0.4, a reduction of about 1% in I_C=O_ values was observed in comparison to neat bitumen. Moreover, a similar trend was observed for bitumen modified with higher amounts of SW_LC_. In comparison, neat bitumen samples containing SW_LC_ and TOA in ratios of 2.5:0.2, 2.5:0.4, and 2.5:0.6 were characterized by I_C=O_ higher by about 79%, 14%, and 75%, respectively. The findings indicate that SW_LC_ is the main donor of polar C=O groups in the investigated bitumen, which is most probably a result of its chemical structure. However, the FTIR spectrum of the SW_LC_ shown in Figure 8 clearly indicates that, before the bitumen modification process, the modifier did not contain C=O groups in its chemical structure. This indicates that, due to high temperature during the bitumen modification process, SW_LC_ molecules are oxidized, which is the main reason for the observed increase in bitumen I_C=O_ values. Moreover, the observed decrease in the I_C=O_ values (in Figure 8a) determined for bitumen modified simultaneously with SW_LC_ and TOA indicates that, during the modification process, chemical reactions probably occur, resulting in a decrease of oxygen content in modifiers and bitumen chemical structures. The basis of these reactions is the uptake of O atoms from oxidized hydrocarbon structures by the corresponding functional groups. The chemical structure of TOA contains amine groups (-NH_2_), which can efficiently capture O from oxidized structures of organic compounds via nucleophilic addition reactions.

A typical chemical reaction occurring during the RTFOT and RTFOT + PAV aging process is oxidation of bitumen, building carbon atoms, and formation of carbonyl groups [71]. The differences between the I_C=O_ values both before and after RTFOT and RTFOT + PAV aging are shown in Figure 8b,c. The results clearly demonstrate the beneficial effect of SW_LC_ on the oxidation reaction of bitumen components. For the reference bitumen, the value of ΔI_C=O_^RTFOT^ was 0.0010, while, after the introduction of SW_LC_ in the amounts of 2.0% and 2.5%, the values of ΔI_C=O_^RTFOT^ were reduced by 19% and 93%, respectively. The favorable effect of SW_LC_ on the oxidation process was also observed for bitumen after PAV aging. In comparison to neat bitumen, the ΔI_C=O_^RTFOT+PAV^ values calculated for bitumen containing 1.5%, 2.0%, and 2.5% SW_LC_ were lower by 17%, 15%, and 13%, respectively. This may be due to the fact that SW_LC_s are low-molecular-weight saturated hydrocarbons that can more easily undergo oxidation reactions while simultaneously protecting the bitumen components. Nevertheless, the beneficial effect of TOA application was observed only for long-term aging. Regarding the neat bitumen after application of 0.2%, 0.4%, and 0.6% TOAs, the values of ΔI_C=O_^RTFOT+PAV^ were lower by 19%, 25%, and 20%, respectively. It clearly indicates that, regardless of amount, TOA inhibits the oxidation reactions of bitumen components during road pavement service but does not inhibit the destructive effects of atmospheric oxygen and high temperature during asphalt mix production. On the other hand, when SW_LC_ and TOA were used synergistically, inhibition of RTFOT aging was observed for their relative ratios of 2.5:0.6; 2.0:0.2; 2.5:0.2, and 1.5:0.6. Compared to unmodified bitumen for these samples, the determined values of ΔI_C=O_^RTFOT^ were lower by about 13.0%, 1.0%, 5.0%, and 16%, respectively. Similarly, the synergistic effect of SW_LC_ and TOA inhibits the RTFOT + PAV aging process. Their application in the ratios of 2.5:0.2, 1.0:0.4, 2.5:0.2, 1.0:0.4, 1.0:0.6, 1.5:0.6, and 2.0:0.6 reduced the values of ΔI_C=O_^RTFOT+PAV^ by about 6.0%, 1.0%, 2.0%, 6.0%, and 6.0%, respectively. The analyzed FTIR spectra show that the efficiency of C=O bond formation was the most effectively inhibited due to the application of SW_LC_ and TOA in the amount of 1.5:0.6. The results clearly show that, in the anti-aging protection of bitumen with a mixture of SW_LC_ and TOA, the appropriate determination of their quantity ratio is crucial. The above data indicate that RTFOT aging is inhibited more effectively by SW_LC_, while PAV aging is inhibited more effectively by TOA. This is because the main destructive factor during RTFOT aging is high temperature, while, during PAV aging, the main factor is the presence of oxygen.

**Figure 9:**
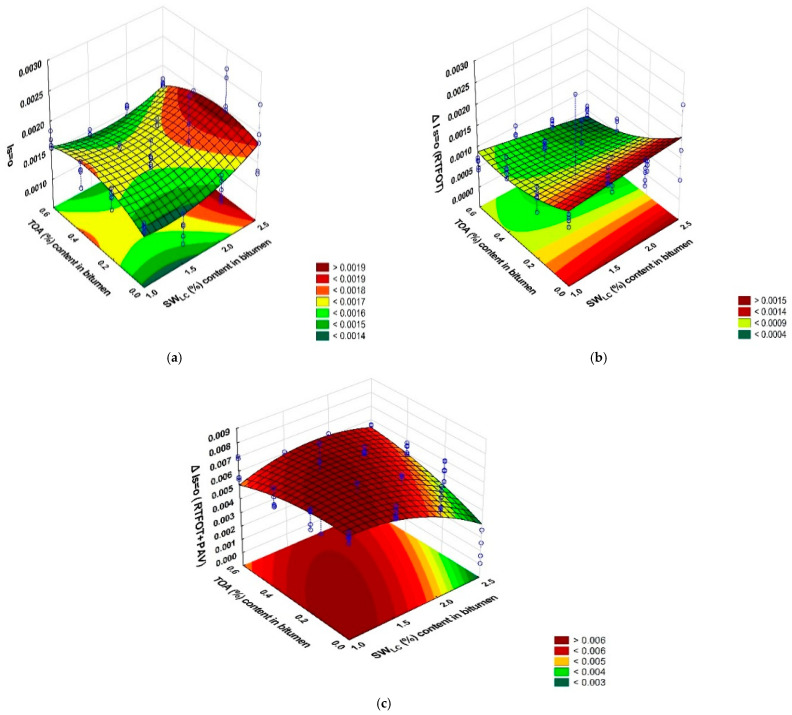
I_S=O_ indexes of bitumen modified with SW_LC_ and TOA before (**a**) and ΔIs_=O_ change after RTFOT (**b**) and RTFOT + PAV (**c**).

Subsequently, the effect of SW_LC_ and TOA on the amount of polar sulfoxide groups (S=O) was analyzed, using the regression models developed in accordance with (14), and the response surface is shown in Figure 9. The significance analysis of each binder component and the interaction between components for the variability I_S=O_, ΔI_S=O_ RTFOT, and ΔI_S=O_ RTFOT + PAV was performed using analysis of variance, ANOVA [68] (Table 8).

The analysis of statistical parameters in Table 8 clearly shows that the SW_LC_ and TOA content had a significant impact on I_S=O_, ΔI_S=O_ RTFOT, and ΔI_S=O_ RTFOT + PAV, as the associated *p*-value is lower than the selected significance level α = 0.05 (*p*-value < 0.05).

Figure 9a clearly shows that SW_LC_ strongly affects the I_S=O_ values than TOA. However, the observed trends are not unambiguous. An increase in I_S=O_ of about 8% compared to the reference bitumen was observed only for bitumen containing 1.5% SW_LC_. For the other modified bitumen containing 1.0%, 2.0%, and 2.5% SW_LC_, the I_S=O_ values were lower by 25%, 17%, and 50%, respectively. Meanwhile, for bitumen modified only with TOA, the calculated I_S=O_ values after the modification process remained unchanged regardless of its amount. For bitumen modified simultaneously with SW_LC_ and TOA in different relative ratios, reduction in I_S=O_ values was observed for bitumen containing 1.0% SW_LC_ and 0.2%, 0.4%, and 0.6% TOA. In these cases determined I_S=O_ values were, respectively, lower about 33%, 33%, and 42%. On the other hand, for bitumen containing the investigated modifiers in ratios of 1.5:0.2, 1.5:0.4, and 1.5:0.6, a reduction in I_S=O_ values, respectively, by 58%, 33%, and 17% was observed. A reduction in I_S=O_ values at very similar levels was also observed for bitumen containing 2.0% SW_LC_ and increasing TOA values. However, the most significant reductions in I_S=O_ values were observed for bitumen modified with SW_LC_ and TOA in ratios of 2.5:0.2; 2.5:0.4; and 2.5:0.6. The reductions of the I_S=O_ index were 83%, 42%, and 42%, respectively.

The bitumen aging process also involves oxidation of S atoms present in its components, i.e., thiophene asphaltene [71]. The effect of this reaction is the formation of polar sulfoxide groups S=O, and the quantitative parameter describing this reaction is the increase in the sulfoxide index (ΔI_S=O_). The values of ΔI_S=O_ calculated for modified bitumen after RTFOT and RTFOT + PAV are seen in Figure 9b,c, respectively. The FTIR spectra analysis indicated the application of SW_LC_ and TOA.

An efficient inhibition occurred in the formation of S=O groups during asphalt mix production. As shown in Figure 9b, the ΔI_S=O_^RTFOT^ of the neat bitumen was 0.0014. Meanwhile, for bitumen modified with SW_LC_ at 2.0% and 2.5%, the ΔI_S=O_^RTFOT^ values decreased by 17% and 49%, respectively. A beneficial effect was also found for bitumen modified only with TOA at 0.2% and 0.4%. For these samples, calculated ΔI_S=O_^RTFOT^ values were, respectively, lower by 20% and 44%, in comparison to the reference bitumen. Similar trends were also observed when SW_LC_ and TOA were simultaneously introduced into the bitumen matrix. A careful analysis of the obtained results indicates that an increase in TOA content with a constant SW_LC_ content of 1.0% resulted in an increase in the anti-aging resistance of the produced modified bitumen. The values shown in Figure 9b,c indicate that an increase in TOA content from 0.2% to 0.6% (with a SW_LC_ content of 1.5%) resulted in a decrease in the S atom oxidation inhibition efficiency. Compared to the reference bitumen, the application of TOA in amounts of 0.2%, 0.4%, and 0.6% resulted in a decrease of ΔI_S=O_^RTFOT^ values by 22%, 16%, and 58%, respectively. In contrast, increasing the SW_LC_ content to 2.0% with a fixed TOA content of 0.4% reduced the ΔI_S=O_^RTFOT^ values by more than a 50% decrease compared to the reference bitumen. In this case, both an increase and decrease in TOA content of 0.2% resulted in a slight increase in the efficiency of inhibition of the S atom oxidation reaction and the formation of polar S=O bonds as indicated by a reduction ΔI_S=O_^RTFOT^ of about 58%. Interestingly, a higher efficiency of S atom oxidation reaction inhibition occurring in the chemical structures of bitumen components was observed for bitumen containing SW_LC_ and TOA in amounts of 2.5% and 0.2%, respectively. In this case, the calculated value of ΔI_S=O_^RTFOT^ was about 81% lower compared to the reference bitumen. In contrast, the increase in TOA amounts of 0.4% and 0.6% resulted in a reduction of ΔI_S=O_^RTFOT^ by 59% and 66%, respectively. These values clearly demonstrate that, in the majority of analyzed modified bitumen, application of SW_LC_ and TOA enhanced resistance of S atoms to destructive atmospheric O_2_ and high temperature within asphalt mixture production. Unfortunately, such favorable trends are not observed for the bitumen after long-term aging (TOA). As Figure 9b,c indicate, an approximate 10% reduction of ΔI_S=O_^RTFOT+PAV^ value was observed only for bitumen modified only with SW_LC_ in an amount of 2.5% FT_LC_. Meanwhile, for bitumen with lower SW_LC_ content (1.0%, 1.5%, 2.0%), an increase of ΔI_S=O_^RTFOT+PAV^ that ranged from 2 to 7% was observed. For bitumen modified only with TOA, its beneficial effect on inhibiting the formation efficiency of polar S=O groups was observed for its amounts of 0.4% and 0.6%. In these cases calculated values of ΔI_S=O_^RTFOT+PAV^ were lower by about 8% and 16%, respectively. As seen in Figure 9b,c, for all bitumen modified simultaneously with both SW_LC_ and TOA, a reduction in ΔI_S=O_^RTFOT+PAV^ values was observed relative to neat bitumen. This indicates that their application enables production of bitumen with higher atmospheric oxygen resistance.

**Figure 10:**
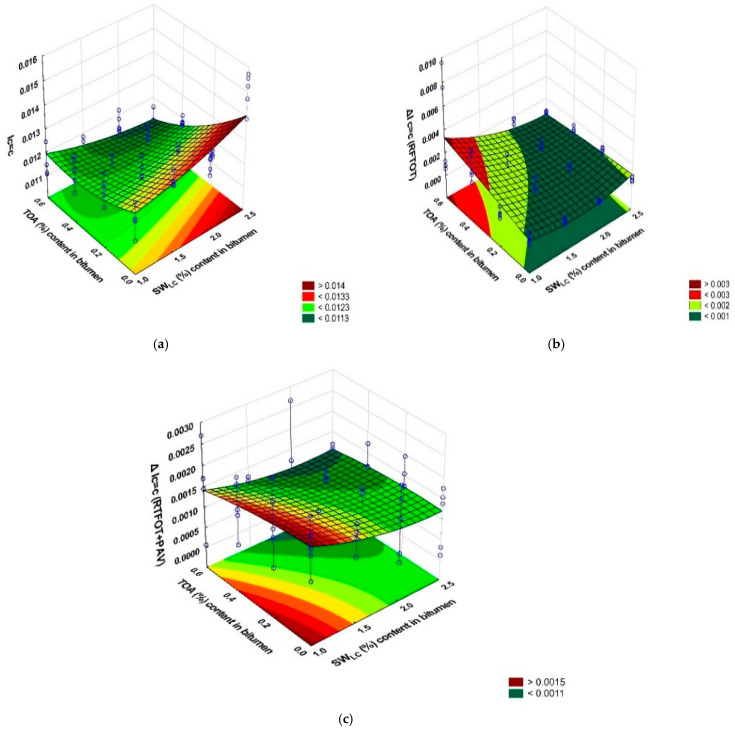
I_C=C_ indexes of bitumen modified with SW_LC_ and TOA before (**a**) and ΔI_C=C_ change after RTFOT (**b**) and RTFOT + PAV (**c**) aging.

Regarding the physicochemical properties of bitumen binders, evaluation of their aromaticity is also very important. It is determined by the presence of benzene rings containing delocalized electrons in their structure, through whose intermolecular π-π interactions directly affect the consistency of the prepared modified bitumen. Subsequently, the effect of SW_LC_ and TOA on the amount of I_C=C_ was analyzed using the developed regression models according to (14), and the response surface is presented in Figure 10. The analysis of the significance of each binder component and the interaction between components for the variability of I_C=C_, ΔI_C=C_ RTFOT, and ΔI_C=C_ RTFOT + PAV was performed using analysis of variance, ANOVA [68]. The results are presented in Table 9.

The analysis of statistical parameters in Table 9 clearly shows that the SW_LC_ and TOA content had a significant effect on I_C=C_, ΔI_C=C_ RTFOT, and ΔI_C=C_ RTFOT + PAV, because the *p*-value associated with it is less than the selected significance level of α = 0.05 (*p*-value < 0.05).

As seen in Figure 10a, the I_C=C_ values of modified bitumen are influenced by both SW_LC_ and TOA. Only for bitumen modified with SW_LC_ in an amount of 2.5% was a 15% increase in I_C=C_ value observed. Meanwhile, road bitumen modification with SW_LC_ in amounts of 1.0%, 1.5%, and 2.0% caused a decrease in I_C=C_ values by 6%, 1%, and 2%, respectively. A reduction of I_C=C_ values was also observed for bitumen containing only TOA. For its amounts of 0.2%, 0.4%, and 0.6%, decreases in I_C=C_ values of 8%, 8%, and 13% were observed, respectively. In addition, similar level changes were observed for bitumen containing increasing amounts of TOA, with a constant SW_LC_ content of 1.0%. Relative to the reference bitumen, a decrease in I_C=C_ index values of about 6%, 7%, and 10% was observed for 0.2%, 0.4%, and 0.6% TOA content, respectively. Slightly more considerable changes in I_C=C_ values were observed for bitumen containing SW_LC_ and TOA in ratios of 1.5:0.2, 1.5:0.4, and 1.5:0.6. In comparison to the reference, the determined I_C=C_ index values for these modified bitumens were lower by 7%, 12%, and 13%, respectively. Changes in I_C=C_ index values at very similar levels were also observed for samples containing 2.0% and 2.5% SW_LC_ with varying TOA contents (0.2–0.6%).

During the short- and long-term aging process, an aromatization process also takes place, which is most susceptible to both cyclohexane structures as well as linear hydrocarbons [20]. It is based on the aromatic ring formation as a result of recombination/rebuilding of the chemical structures within the hydrocarbons that build bitumen. To quantitatively describe the aromatization process, the I_C=C_^RTFOT/RTFOT+PAV^ index is commonly employed, which is determined based on the band surface area located at a wavenumber of 1600 cm^−1^ attributed to the stretching vibrations of the unsaturated bonds in the benzene ring. The I_C=C_, ΔI_C=C_^RTFOT^, and ΔI_C=C_^RTFOT+PAV^ values shown in Figure 10a–c indicate that, in the majority of investigated modified bitumen, applications of WS_LC_ and TOA do not affect the aromatization process. The analysis suggests that a reduction in ΔI_C=C_^RTFOT^ of only about 1.8% compared to neat bitumen was observed only for a sample containing 0.4% TOA. In contrast, a reduction in ΔI_C=C_^RTFOT^ at a similar level among SW_LC_-modified samples was observed only for its content of 2%. As for bitumen containing both SW_LC_ as well as TOA, a reduction in ΔI_C=C_^RTFOT^ was observed only for bitumen containing them at ratios of 1.0:0.6; 2.0:0.4, 2.0:0.6, and 2.5%:0.4. The observed reduction in ΔI_C=C_^RTFOT^ values was 26%, 17%, 2.3%, and 6.5%, respectively. As shown in Figure 10b,c, both SW_LC_ and TOA also do not effectively inhibit the aromatization process occurring during the modified bitumen long-term aging. The determined indices suggest that this process is inhibited only for bitumen containing 0.6% TOA, as well as 2.0% and 2.5% SW_LC_. In these cases, a reduction in ΔI_C=C_^RTFOT+PAV^ of about 88%, 20%, and 88%, respectively, was observed. On the other hand, among the bitumen modified simultaneously with WS_LC_ and TOA, the inhibition of the aromatization process occurring within TOA aging was observed for their mutual ratio of 2.0:0.4; 2.0:0.6, and 2.5:0.4. In these cases, a decrease in ΔI_C=C_^RTFOT+PAV^ values was observed by about 4%, 20%, and 16%, respectively.

In conclusion, the spectroscopic analyses show that modification of bitumen with SW_LC_ and TOA in appropriate mass ratios makes it possible to effectively inhibit the chemical changes occurring during its short- and long-term aging. The determined ratios clearly indicate that, in SW_LC_ and TOA, it is possible to reduce the efficiency of the oxidation reaction of C atoms and S atoms and the aromatization process of the aliphatic components of bitumen binders.

### 3.4. Optimization of WMA Additives in Bitumen

**Figure 11:**
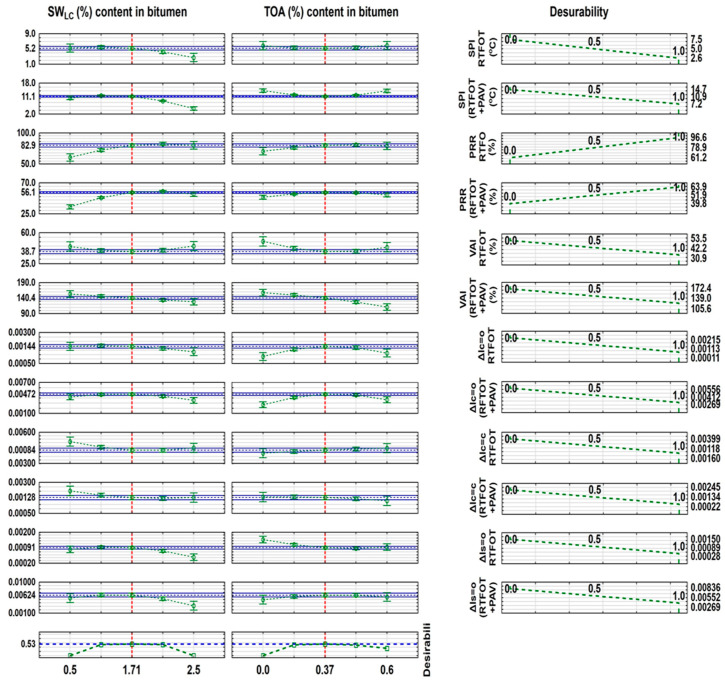
Optimization of the amount of WMA additives (SW_LC_ and TOA) in bitumen in terms of aging characteristics.

The physical, mechanical, and rheological properties of asphalt are determined by its chemical composition. The aging process, during which these properties change, also plays a significant role. The results obtained during the tests indicated the amounts of SW_LC_ and TOA in bitumen that ensured the optimal value for a single parameter. Therefore, in order to achieve a compromise solution, the composition of the designed material—bitumen with WMA additives (SW_LC_ and TOA)—was optimized using the desirability function (D) method in terms of its aging characteristics in order to determine its final most advantageous properties. An analysis of the aging characteristics of bitumen was carried out, taking into account the minimization of SPI_RTFOT_, SPI_RTFOT+PAV_, VAI_RTFOT_, VAI_RTFOT+PAV_, as well as the carbonyl index (I_C=O_), sulfoxide index (I_S=O_), and aromaticity index (I_C=C_) determined after the RTFOT and RTFOT + PAV aging processes, and the maximization of PRR_RTFOT_, PRR_RTFOT+PAV_, which ensure the durability of the asphalt pavement. In order to optimize the amount of WMA additives in the binder, the desired property desirability functions [67,70] were used, which were analyzed using the Statistica software 13.3 [68]. The results of the analysis are presented in graphical form in Figure 11, which also shows the trend of favorable changes for the analyzed aging characteristics of the binder.

Based on the desirability function determined using the Statistica program 13.3 [68], the optimal SW_LC_ content (solution in grid nodes) was set at 1.71 *w*/*w* and TOA at 0.37 *w*/*w* in bitumen before and after its modification with WMA additives. Taking into account the scope and process of dosing the components (WMA additives) into the bitumen during the experiment, the recommended amount of SW_LC_ was set at 1.50% *w*/*w* and TOA at 0.4 *w*/*w* in bitumen. It should be noted that the specified WMA additive contents are a compromise between the most favorable individual values for the tested properties and may differ from those specified for a single property.

Based on the analysis of Figure 11 showing the desirability functions of D, it can be concluded that a deviation of approximately ±0.2% SW_LC_ or ±0.1% TOA causes a maximum 5% decrease in desirability functions, which allows for some flexibility in the process.

#### 3.4.1. Physical and Mechanical Characteristics of the Binder for the Optimal Amount of Additives

**Figure 12:**
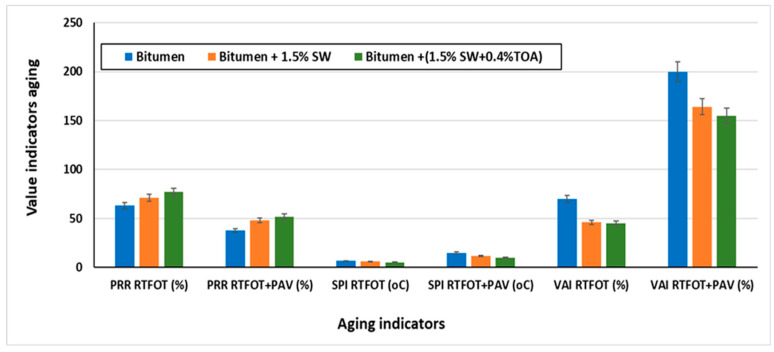
Aging indices PRR, SPI, and VAI of bitumen without and optimal amount of WMA additives (1.5% SW + 0.4%TOA) and bitumen with 1.5% SW_LC_.

A very important element of the analysis of WMA additive optimization was to compare the PRR, SPI, and VAI aging indices of bitumen with and without WMA additives after the RTFOT and RTFOT + PAV aging processes. For comparison purposes, bitumen with 1.5% SW_LC_ additive was also analyzed. The results of the comparison are presented in Figure 12.

By analyzing the PRR, SPI, and VAI aging indices, it can be clearly concluded that the use of WMA additives (1.5% *w*/*w* SW_LC_ and 0.4% *w*/*w* TOA) in the optimal amount has a favorable effect on reducing the impact of RTFOT and RTFOT + PAV aging on the properties of the bitumen containing them compared to bitumen without additives or only with 1.5% SW_LC_. The PRR values after RTFOT and RTFOT + PAV aging for bitumen with WMA additives show a more favorable trend of change than for bitumen without WMA additives or with only 1.5% SW_LC_. A similar trend is found when analyzing the SPI of bitumen with WMA additives. The most significant beneficial effect of WMA additives in bitumen can be observed by analyzing the VAI index after RTFOT and especially RTFOT + PAV aging. The effect of WMA additives on reducing bitumen aging in this case is very significant. Such beneficial anti-aging effects of WMA additives in bitumen can only be explained by the effect of the synergy occurring between them.

#### 3.4.2. FTIR for the Recommended Amount of WMA Additives in Bitumen

**Figure 13:**
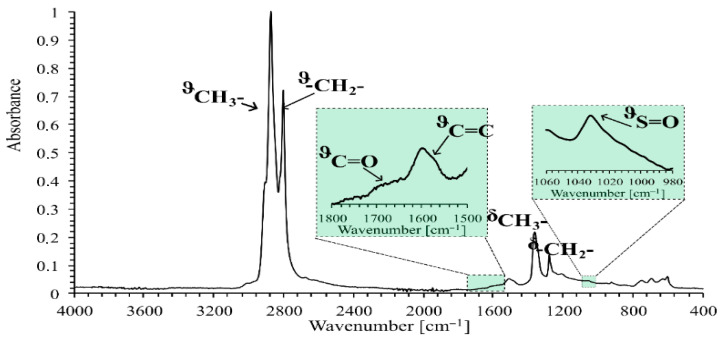
FTIR spectra of bitumen modified with SW_LC_ and TOA in amounts of 1.5% and 0.4%, respectively.

The spectrogram of 50/70 bitumen with dosed optimum additive contents of SW_LC_ and TOA after RTFOT and RTFOT + PAV aging is shown in Figure 13.

**Figure 14:**
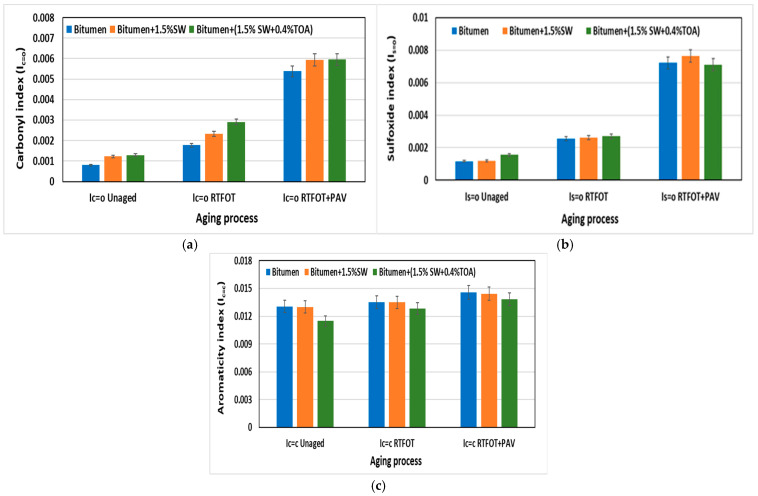
Effect of RTFOT and RTFOT + PAV aging on bitumen, bitumen + 1.5% SW and with optimal amount of WMA additives in terms of (**a**) carbonyl index (I_C=O_), (**b**) sulfoxide index (I_S=O_), and (**c**) aromaticity index (I_C=C_).

The values of carbonyl index (I_C=O_), sulfoxide index (I_S=O_), and aromaticity (I_C=C_) for bitumen, bitumen with 1.5% *w*/*w* SW_LC_, and with the optimal amount of WMA additives are shown in Figure 14.

Analysis of the test results shown in Figure 14a shows that the bitumen has the lowest value of the carbonyl index I_c-o_ = 0.0008. The application of 1.5% SW_LC_ additive to the bitumen causes an increase in the carbonyl index I_c-o_ = 0.0013, and the application of the optimal amount of WMA additives to the bitumen causes a further increase in its I_C=O_ = 0.0013. As a result of RTFOT and RTFOT + PAV aging, an increase in the carbonyl index of the tested bitumen is observed with the trend occurring as for unaged bitumen. It should be noted that the aging of RTFOT + PAV causes a very significant increase in the carbonyl index of the tested bitumens, reaching more than four times its value for unaged bitumens, which indicates its significant effect on the binder.

Unaged bitumen without WMA additives has a sulfoxide index of I_s-o_ = 0.0011 (Figure 14b). The application of a 1.5% SW_LC_ additive to the bitumen results in a slight increase with I_s-o_ = 0.0012, and this tendency intensifies when the optimum amount of WMA additives is applied to the bitumen I_s-o_ = 0.0016. RTFOT aging results in an increase in the sulfoxide index value of up to two times compared to its value for unaged bitumen. RTFOT + PAV aging, on the other hand, continues this trend, and the sulfoxide index value I_s-o_ increases by almost seven times. Analysis of the test results shown in Figure 14c allows us to conclude that the aromaticity index of the unaged bitumen is I_c-c_ = 0.0131. However, the application of the addition of 1.5% SW_LC_ to the bitumen lowers it to I_c-c_ = 0.0130, and the application of the optimal amount of WMA additives perpetuates this trend, as I_c-c_ = 0.0115. A similar relationship is observed after the application of RTFOT and RFTOT + PAV aging for the tested bitumen. This shows that, as a result of synergy, SW_LC_ and TOA in optimal amounts have a favorable effect on the aromaticity index I_C=C_.

In conclusion, it can be said that the application of the optimal amount of WMA additives to the bitumen before aging and after the RTFOT and RTFOT + PAV aging process favorably affects its aromaticity index, which also has a significant beneficial effect on its rheological properties.

#### 3.4.3. Low-Temperature Characteristics of Bitumen for Optimal WMA Additive Compactness

**Figure 15:**
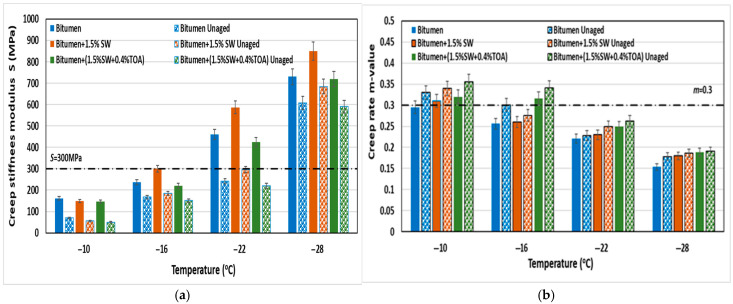
BBR test results: (**a**) creep stiffness S of bitumen; (**b**) creep rate m-value of bitumen.

The changes in the modulus of creep stiffness S and the value of creep rate m of bitumen at different temperatures are shown in Figure 15.

The parameter S represents stiffness at low temperature, and, as the temperature decreased, a lower value of S corresponded to greater elasticity of the bitumen. On the other hand, the value of m reflected the ability of the bitumen to relax stress at low asphalt temperatures, and the increase in its value with decreasing temperature indicates its high resistance in the negative temperature range [72]. The SHRP specification stated that, in order to ensure the low-temperature properties of the binder, the S-values of the bitumen at the design temperature should be less than 300 MPa, and the m-values should be greater than 0.3 [73].

Tests were performed on a series of nine samples for each additive combination. Control bitumen, bitumen with 1.5% SW_LC_, and bitumen with the optimal amount of WMA additives were tested before aging and after RTFOT + PAV aging according to the EN 14771 procedure. Analysis of the test results showed that, after aging, there is an increase in the S-value with decreasing temperature, while the m-value decreased. This indicates that the RTFOT + PAV aging to which the binders were subjected during sample preparation for the BBR test caused a deterioration in the low-temperature elasticity and stress relaxation properties of the bitumen. This effect is caused by the volatilization of lightweight binder components due to oxygen–thermal interaction during the preparation of bitumen for testing, and the consequence is an increase in stiffness at low temperatures. In the temperature range of −10 °C, it can be unequivocally stated that the creep stiffness modulus S of bitumen with WMA additives in the optimum amount is lower than that of bitumen without additives or bitumen with the addition of1.5% SW_LC_, regardless of whether the binder was aged or not. On the other hand, in the temperature range from −16 °C to −28 °C, bitumen with the optimal amount of WMA additives has the lowest S-value, followed by bitumen without additives and bitumen with 1.5% SW_LC_ added. In the temperature range from −16 °C to −22 °C, the S = 300 MPa criterion is met for all unaged binders and for aged binders (PAV) in the range from −10 °C to −22 °C. In contrast, at −28 °C, all binders do not meet the S = 300 MPa criteria. It should be noted that this is an extreme temperature, which is now practically not ascendant from the winter period in Europe. Therefore, a bitumen taking the optimal amount of SW_LC_ and TOA will be more resistant to low-temperature cracking than a control bitumen or one with only 1.5% SW_LC_. It should be noted that the change in the S-parameter of bitumen with WMA additives in the range of negative temperatures analyzed is more favorable than that of control bitumen or bitumen with the addition of 1.5% SW_LC_.

A similar favorable trend as for the S parameter is observed for the creep rate m comparing tests of bitumen without WMA additives, bitumen with 1.5% SW_LC_ additives, and with WMA additives in the optimal amount. The value of the parameter m of bitumen with WMA additives is the highest compared to bitumen without additives and with the addition of 1.5% SW_LC_ over the entire range of negative temperatures. Over the entire range of negative temperatures, the value of the creep rate m of bitumen with WMA additives is more favorable compared to the other binders. This indicates that the asphalt containing 1.5% SW_LC_ and 0.4% TOA is much more capable of stress relaxation at low temperature with respect to the other binders.

Bitumen with WMA additives (1.5% *w*/*w* SW_LC_ and 0.4% *w*/*w* TOA) showed the best cracking resistance as a result of their synergy, which is most likely also due to the effect of the aromatic compound (I_C=C_), which results in increased relaxation capacity at low temperatures.

#### 3.4.4. MSCR Analysis

**Figure 16:**
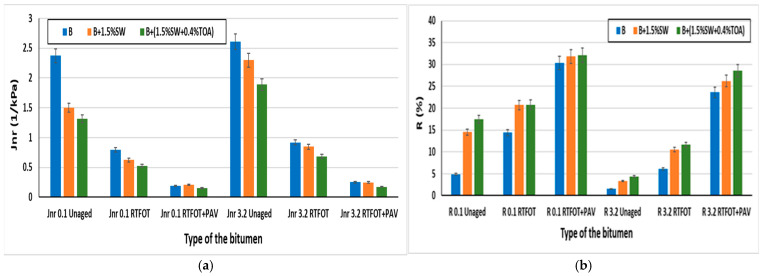
MSCR parameters for binders at two stress levels (0.1 kPa) and 3.2 kPa): (**a**) Jnr (1/kPa), (**b**) R (%) for bitumen, bitumen + 1.5%SW and bitumen with optimal SW and TOA of 1.5% and 0.4%, respectively.

At present, the prediction of the effect of the binder and especially the polymer-modified binder [63] on the formation of permanent deformation of asphalt pavement is evaluated on the basis of the MSCR test. In order to accurately reflect actual conditions, the test used short-term aged samples corresponding to the performance of the binder in a newly constructed asphalt layer and long-term aged samples corresponding to its performance in the pavement over a long period of service. For control purposes, samples of the new binder were also analyzed. The tests used bitumen 50/70, bitumen 50/70 with the addition of 1.5% SW_LC_, and with the optimal composition of SW_LC_ and TOA. The results of the tests performed were two parameters R and Jnr at stress levels of 0.1 (1/kPa) and 3.2 (1/kPa). It is assumed that bitumen has a lower susceptibility to rutting, the lower the parameter Jnr and the higher the parameter R. Tests were performed on at least five samples. The average values of the binder’s MSCR test results characterized by the Jnr and R parameters are shown in Figure 16.

The results, shown in Figure 16, indicate that, compared to the Jnr and R parameter values at two stress levels for the reference bitumen, bitumen with 1.5% SW_LC_ and bitumen with optimal SW_LC_ and TOA additives were, in all cases, less susceptible to rutting both before and after aging. Recovery (R) was higher, while irreversible creep susceptibility (Jnr) was lower at both stress levels compared to the 50/70 reference bitumen and bitumen with 1.5% SW_LC_ additive. The results corroborate previous findings on bitumen modification with such polymers as styrene-butadiene-styrene (SBS) or vinyl acetate (EVA) [74,75], PANI-NF nanofibers [19] as well as the use of various organic or mineral additives.

The observed increase in R recovery and decrease in irreversible creep susceptibility Jnr was due to the type of binder tested (plain and with the addition of 1.5% SW_LC_) and the modification of the binder with SW_LC_ and TOA. The favorable change in R and Jnr characteristics is the result of less aging that the bitumen with SW_LC_ and TOA underwent and thus has a more elastic character, analogous to other types of modifiers that form a spatial network in the bitumen [76]. Bitumen with optimal SW_LC_ and TOA has more than twice the R-value and twice the Jnr-value compared to control bitumen at a stress level of 0.1 (1/kPa). This trend occurs equally at a stress level of 3.2 (1/kPa) but more intensively. The aging process does not change the tendency of recovery behavior R or irreversible creep susceptibility Jnr of the tested bitumen. In bitumen samples with WMA additives after PAV aging, the improvement in resilient rebound R0.1 was maintained at 75–80% after RTFOT. Therefore, this suggests good durability of such a modified bitumen composition. The results obtained indicate that the use of asphalt with the optimal amount of SW_LC_ and TOA in the composition of the mineral–asphalt mixture can provide it with increased resistance on the basis of permanent deformation compared to the use of bitumen 50/70 or only with the addition of 1.5% SW_LC_ [76,77].

#### 3.4.5. Approximation of CAM Model Parameters

Tests on the rheological properties of bitumen (composite shear modulus G*, phase shift angle δ) in a DSR dynamic shear rheometer were determined at temperatures from 10 °C to 90 °C in 10 °C steps at frequencies from 0.16 to 16 Hz under controlled deformation conditions according to EN 14770. The correct determination of the composite shear modulus G* and phase shift angle δ is very important because these parameters are used in analytical methods and pavement design procedures.

The parameters of the CAM model were estimated using the nonlinear least squares method. The optimization performed to find the best fit between the experimental and model data was supported by the simultaneous use of Hooke–Jeeves and Quasi-Newton solvers [78,79]. As a result, a set of CAM model parameters was obtained (Table 10), based on which composite dynamic modulus |G*| and phase shift angle δ were determined by analytical methods according to the study of [79].

**Figure 17:**
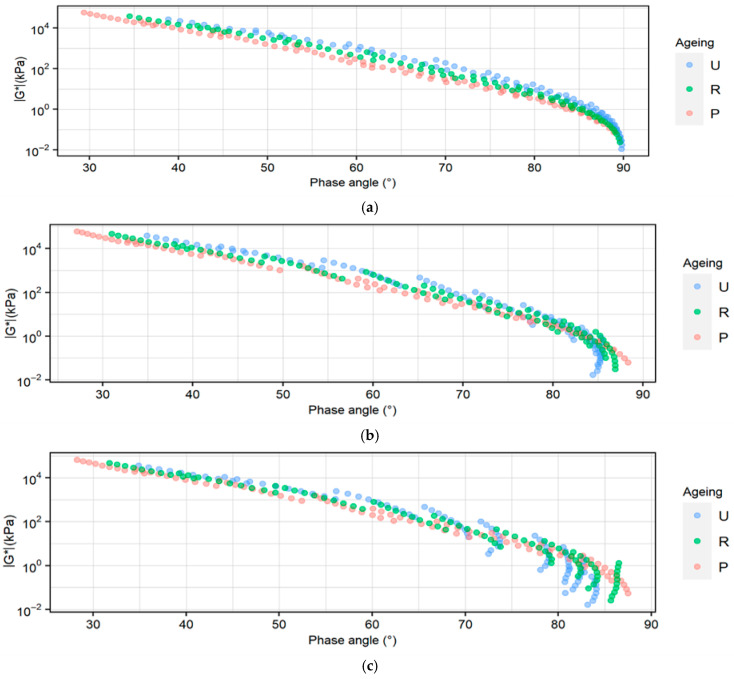
Black diagrams of the investigated bitumens: (**a**) bitumen—B, (**b**) bitumen + 1.5%SW, (**c**) bitumen + (1.5%SW + 0.4%TOA). Ageing: U—unaged, R—RTFOT, and P—RTFOT + PAV.

Figure 17 presents the results of the oscillatory tests obtained using the dynamic shear rheometer to evaluate the dynamic moduli and phase angles of the investigated binders in a wide range of temperatures and loading frequencies in the linear viscoelastic domain. Based on the presented Black curves and their continuity, it can be assessed that the investigated binders are mostly thermorheologically simple, and time–temperature superposition can be applied [39].

Based on the analysis of the test results (Figure 17a–c), it can be concluded that the use of 1.5% SW_LC_ and WMA additives (SW_LC_ and TOA) in the reference binder causes a decrease in the phase shift angle δ. At the same time, it is more pronounced when 1.5% SW_LC_ and 0.4% TOA are used in the binder. This indicates the dominance of the elastic part G’ of the dynamic modulus in the binder. In unaged binders, the synthetic wax content of SW_LC_ and TOA will contribute to the increase in the dynamic modulus value |G*|. In addition, in the case of asphalt with the addition of SW_LC_ and TOA, a characteristic curvature can be observed, which is greater than in the case of using only SW_LC_, indicating the favorable rheological properties of this binder in terms of its performance. Thus, it can be concluded that, at high test temperatures, the level of elastic recurrence of asphalt with SW_LC_ and TOA will increase. The RTFOT aging process causes a decrease in the phase shift angle in asphalt with SW_LC_ and with the addition of SW_LC_ and TOA to a level close to the reference asphalt. On the other hand, after the RTFOT + PAV process, the phase shift angle increases and is almost equal to the phase shift angle of the control bitumen. The difference in the phase shift angle between the reference asphalt and the PAV-aged bitumen with the addition of 1.5% SW_LC_ and 1.5% SW_LC_ and 0.4% TOA is about 3.5° and 2.0°, respectively (Figure 14b,c). In summary, the increase in dynamic modulus |G*| of bitumen with the addition of 1.5% SW_LC_, especially with the addition of 1.5% SW_LC_ and 0.4% TOA to the reference bitumen, will result in a mineral–asphalt mixture made with this type of binder with high resistance to permanent deformation.

**Figure 18:**
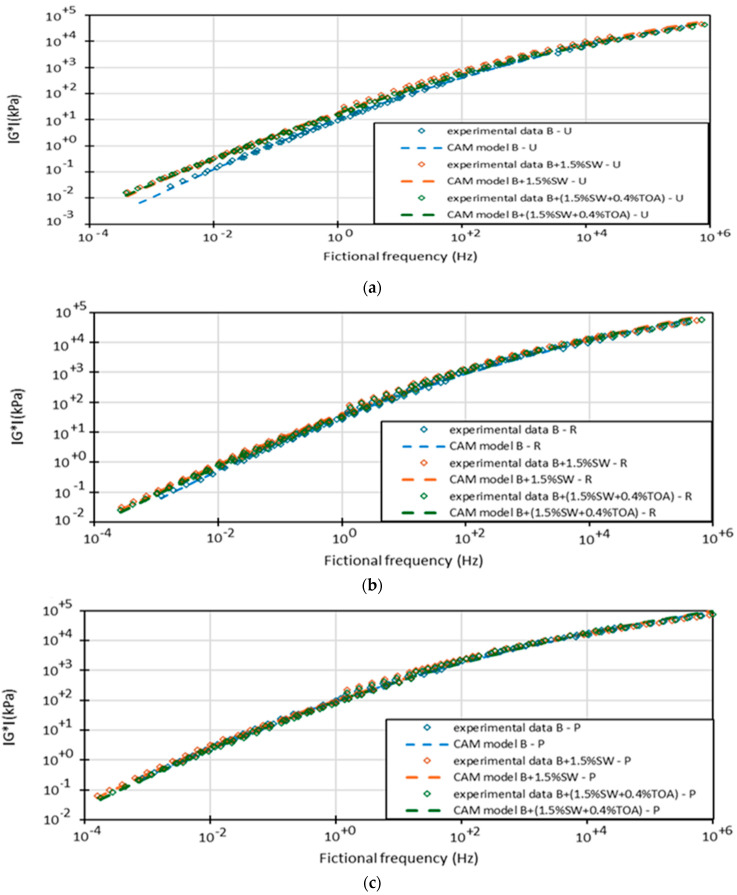
Dynamic modulus |G*| master curves of the bitumen—B, bitumen B + 1.5% SW_LC_, and modification of bitumen B+ (1.5% SW_LC_ + 0.4% TOA) after aging: (**a**) unaged—U, (**b**) RTFOT—R, (**c**) RTFOT + PAV—P. Aging: U—unaged, R—RTFOT, and P—RTFOT + PAV.

Dynamic modulus master curves of the evaluated bitumens were constructed using the CAM model based on their approximate thermorheological simplicity (shown graphically together with the shifted data in Figure 18. The parameters of the fitted models defining the above-mentioned master curves are shown in Table 10.

Determination coefficient R^2^ was more than 92% and confirmed the exact fit of the CAM model curve to the experimental results at the test temperature.

Here, it can be observed that the processes applied and the additives used with the bitumen significantly affected their rheological behavior. The aging stress induced on the tested materials resulted in consistent decreases in the crossover frequency (f_c_) parameter in the obtained rheological models, which indicates decreases in the phase angle and more pronounced elastic behavior of the binders. This is consistent with the general trend observed by researchers using other types of asphalt additives [80,81]. Similar results were obtained in regard to the rheological index (R_c_), which in most cases increased with the introduction of additional aging, indicating broadening of the relaxation spectrum due to the domination of elastic properties of the binders in a larger range of temperatures and loading frequencies. Significant observations in this regard include the fact that both the crossover frequency and rheological indices of the bitumen containing the WMA additives (1.5% SW_LC_ + 0.4% TOA) were much less affected by aging compared to the non-modified bitumen and bitumen only with 1.5% SW_LC_. Particularly, the effect was most pronounced in the RTFOT-aged samples, but, even after the PAV aging, the effect was not trivial.

**Figure 19:**
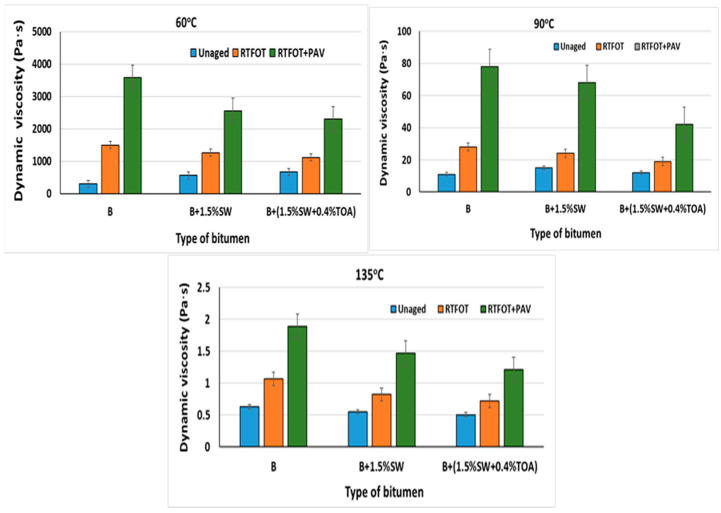
Dynamic viscosity of the investigated bitumen measured in three temperatures (60 °C, 90 °C, 135 °C).

Figure 19 shows the results of dynamic viscosity measurements performed at three distinct temperatures: 60 °C relating to high pavement temperature, 90 °C approximated to the phase shift in the synthetic wax, and 135 °C in the processing temperature region of the asphalt mixture.

Regarding the dynamic viscosity measurements at all temperatures, the imposed aging had a significant role in shaping the differences seen between the investigated bitumen. The most pronounced effect among those observed occurred after RTFOT + PAV aging. The introduction of the 1.5% SW_LC_ additive and both WMA additives (1.5% SW_LC_ and 0.4% TOA) into the asphalt resulted in a decrease in the dynamic viscosity of the aged RTOFT + PAV binder, which was as high as approx. 50% at 90 °C for bitumen with WMA additives. On the other hand, the RTFOT process will significantly affect the variation in the viscosity values of the tested binders compared to RFTOT + PAV aging.

Therefore, the use of WMA additives in the optimal amount to the bitumen will more favorably affect the properties of the asphalt mixture than the use of control bitumen.

## 4. Conclusions

In order to meet the environmental requirements for road construction in terms of guaranteeing the service life of asphalt surfaces, special attention is paid to ensuring high requirements in terms of bitumen properties, in particular regarding the limitation of their adverse changes as a result of the short-term and long-term aging process. For this purpose, the addition of synthetic wax and of tall oil amidopolyamine to bitumen was evaluated in terms of their effects after laboratory short and long-term aging processes. Based on the results of this research, the following recommendations were formulated:

1. The use of synthetic wax (SW_LC_) and tall oil amidopolyamine (TOA) in optimal proportions of 1.5% *w*/*w* and 0.4% *w*/*w* has a positive effect on the physical and mechanical properties of the bitumen. There was a 22% reduction in penetration at 25 °C, a 28% increase in softening temperature, and an 18% reduction in viscosity at 135 °C.
2. The synergy of synthetic wax and tall oil amidopoliamine ensures the negative impact of short- and long-term aging on penetration at 25 °C, which is characterized by an increase in the PRR_RTFOT_ index by 22% and PRR_RTFOT+PAV_ by 37%. The softening temperature is characterized by a 20% reduction in the SPI_RTFOT_ index and a 33% reduction in the SPI_RTFOT+PAV_ index, and the dynamic viscosity is characterized by a 36% reduction in the VAI_RTFOT_ index and a 23% reduction in the VAI_RTFOT+PAV_ index.
3. Synthetic wax and tall oil amidopolyamine have a positive effect within the recommended dosage range and also allow the original chemical structure of the bitumen components to be preserved by inhibiting oxidation reactions and the formation of polar C=O, S=O, and C=C bonds after both short-term and long-term aging. This is particularly evident in the case of a decrease in I_C=C_ after RTFOT and RTFOT + PAV aging of bitumen with optimal amounts of WMA additive of approximately 10% compared to control bitumen.
4. The synergistic effect of SW_LC_ and TOA has a positive impact on the low-temperature properties of the binder, i.e., ensuring a reduction in the temperature range from −10 °C to −28 °C, a reduction in the creep stiffness modulus S before aging in the range of 4% to 19% and after aging in the range of 3% to 28%, and an increase in the creep rate m before aging from 9% to 22% and after aging from 2% to 15%. The advantageous rutting properties of the Jnr 0.1 binder after RTFOT aging changed by about 36%; after RTFOT + PAV by about 21% compared to the control asphalt and Jnr 3.2; after RTFOT aging by about 24%; after RTFOT + PAV by about 30% and R 0.1 and R 3.2 by about 44% and 90%; and after RTFOT and after RTFOT + PAV by 6.5% and 21%, respectively, compared to the control asphalt, as well as the dynamic shear properties (|G*|, sin(δ)) in the oscillatory model, which are also ensured.
5. The introduction of WMA additives (SW_LC_ and TOA) to bitumen improved its rheological properties in the unaged state and after short-term aging, increasing the measured phase angles by 3° and 5°, respectively, thus resulting in additional structuring of the bitumen.
6. The assessment of rheological properties using the CAM model showed that WMA additives (SW_LC_ and TOA) significantly inhibited changes in the frequency of returns fc and the rheological index Rc, causing bitumen aging, thus making the binders more stable throughout the entire service life of the pavement.
7. The effect on the flow characteristics of bitumen at higher temperatures (60 °C and above), quantified by dynamic viscosity measurements in a range of more than two times, was more pronounced in samples subjected to long-term aging, confirming the significance of the anti-aging effect of the investigated formulation.
8. The synergistic effect of 1.5% synthetic wax and 0.4% tall oil amidopolyamines most effectively inhibits the destructive aging processes of road bitumens.

Bitumen containing optimal amounts of SW_LC_ and TOA, 1.5% and 0.4%, respectively, will have a significant impact on the technological process and quality of the asphalt mixture produced using WMA technology. A binder with optimal amounts of SW_LC_ and TOA additives allows for the production of asphalt mixture at a temperature of ≤145°, thus enabling a reduction in its production temperature by 20 °C to 30 °C in the asphalt concrete plant. At the same time, there will be no deterioration in its properties. On the other hand, slowing down the asphalt aging process will ensure the durability of the asphalt pavement over its long exploitation period. Lowering the production temperature of the asphalt mixture will contribute to the reduction in greenhouse gases, which will reduce the negative aspects of the asphalt concrete plant’s operations and improve environmental protection.

## Figures and Tables

**Table 1:** Basic properties of the base 50/70 bitumen.

Property	Standard	Unit	Mean Value
Penetration at 25 °C	EN 1426 [48]	0.1 mm	60.1
Softening point T_R&B_	EN 1427 [49]	°C	49.6
Fraass breaking point	EN 12593 [50]	°C	−16.0
Dynamic viscosity at 60 °C	EN 12596 [51]	Pa·s	370.5
Dynamic viscosity at 135 °C	EN 12596 [51]	Pa·s	0.617

**Table 2:** Characteristics of the additives used in research [52,53].

Property	Unit	Additive SW_LC_	Additive TOA
Appearance	-	Solid pellets, white or yellowish	Viscous liquid, brown
Flash point	°C	285	>218
Solidification point	°C	95	<0
Density at 25 °C	Mg/m^3^	0.9	
Density at 20 °C	Mg/m^3^		0.88–0.98
Viscosity at 135 °C	Pa·s	12	
Viscosity at 20 °C Brookfield spindle SC 4-183	m Pas		3000
Molecular weight	g/mol	ca. 1000	

**Table 3:** Fit function parameters for the Pen25 (0.1 mm), T_R&B_ (°C), and η_135_ (Pa·s).

Effect	Pen_25_ (0.1 mm); R^2^ = 0.929; MSE = 4.985			T_R&B_ (°C); R^2^ = 0.967; MSE = 0.776			η_135_ (mPa·s); R^2^ = 0.951; MSE = 47.924		
	RC	SE	*p* Value	RC	SE	*p* Value	RC	SE	*p* Value
Intercept	98.260	2.295	0.000	45.559	0.905	0.000	620.031	7.118	0.000
(1) SW_LC_ (L)	−59.804	2.663	0.000	6.767	1.050	0.000	−70.736	8.259	0.000
SW_LC_ (Q)	13.205	0.744	0.000	0.802	0.293	0.007	5.288	2.307	0.023
(2) TOA (L)	22.082	3.907	0.000	2.694	1.541	0.082	−116.35	12.114	0.000
TOA (Q)	−36.388	4.651	0.000	6.434	1.835	0.000	16.986	14.422	0.240
1Lx2L	7.224	1.488	0.000	−4.236	0.587	0.000	21.565	4.6151	0.000

Where Q—quadratic term; L—linear term; RC—regression coefficient; and SE—std. error.

**Table 4:** Fit function parameters for the PRR_RTFO_ and PRR_RTFOT+PAV_.

Effect	PRR_RTFOT_ (%); R^2^ = 0.560; MSE = 33.354			PRR_RTFOT+PAV_ (%); R^2^ = 0.700; MSE = 11.023		
	Reg. Coeffic.	Std. Error	*p* Value	Reg. Coeffic.	Std. Error	*p* Value
Intercept	34.6172	5.93828	<0.001	7.3427	3.413788	0.033
(1) SW_LC_ (L)	42.1955	6.89019	<0.001	45.1188	3.961017	<0.001
SW_LC_ (Q)	−10.1663	1.92511	<0.001	−10.8946	1.106706	<0.001
(2) TOA (L)	15.2219	10.10685	0.134	13.9837	5.810204	0.017
TOA (Q)	−25.3985	12.03196	0.036	−14.3825	6.916910	0.039
1Lx2L	7.2852	3.85023	0.060	0.0347	2.213411	0.987

**Table 5:** Fit function parameters for the SPI_RTFOT_ and SPI_RTFOT+PAV_.

Effect	SPI_RTFOT_ (°C); R^2^ = 0.501; MSE = 0.741			SPI_RTFOT+PAV_ (°C); R^2^ = 0.801; MSE = 0.743		
	Reg. Coeffic.	Std. Error	*p* Value	Reg. Coeffic.	Std. Error	*p* Value
Intercept	7.026	0.886	<0.001	9.005	0.886	<0.001
(1) SW_LC_ (L)	0.612	1.028	0.552	7.027	1.028	<0.001
SW_LC_ (Q)	−0.857	0.2873	0.003	−2.833	0.287	<0.001
(2) TOA (L)	−9.731	1.508	<0.001	−11.487	1.508	<0.001
TOA (Q)	3.869	1.795	0.032	14.643	1.795	<0.001
1Lx2L	4.231	0.574	<0.001	1.2627	0.574	0.029

**Table 6:** Fit function parameters for the VAI_RTFOT_ and VAI_RTFOT+PAV_.

Effect	VAI_RTFOT_ (%); R^2^ = 0.413; MSE = 31.817			VAI_RTFOT+PAV_ (%); R^2^ = 0.525; MSE = 171.467		
	Reg. Coeffic.	Std. Error	*p* Value	Reg. Coeffic.	Std. Error	*p* Value
Intercept	58.374	5.799	<0.001	170.289	13.4641	<0.001
(1) SW_LC_ (L)	−15.906	6.729	0.019	0.434	15.622	0.977
SW_LC_ (Q)	4.810	1.880	0.011	−4.862	4.364	0.267
(2) TOA (L)	−23.668	9.871	0.017	−73.160	22.915	0.001
TOA (Q)	36.1730	11.751	0.002	−31.706	27.280	0.247
1Lx2L	−4.236	3.760	0.261	22.408	8.729	0.011

**Table 7:** Fit function parameters for the I_C=O,_ ΔI_C=O_ RTFOT, and ΔI_C=O_ RTFOT + PAV.

Effect	I_C=O_; R^2^ = 0.246; MSE = 0.0000002			ΔI_C=O_ RTFOT; R^2^ = 0.317; MSE = 0.0000002			ΔI_C=O_ RTFOT + PAV; R^2^ = 0.493; MSE = 0.000003		
	RC	SE	*p* Value	RC	SE	*p* Value	RC	SE	*p* Value
Intercept	0.00216	0.0007	0.002	0.00104	0.0006	0.088	0.00300	0.0007	0.000
(1) SW_LC_ (L)	−0.00133	0.0007	0.095	0.00033	0.0007	0.635	0.00159	0.0008	0.062
SW_LC_ (Q)	0.00051	0.0002	0.021	−0.00024	0.0002	0.223	−0.0008	0.0002	0.005
(2) TOA (L)	−0.00042	0.0011	0.709	0.00193	0.0010	0.062	0.00286	0.0012	0.023
TOA (Q)	0.00285	0.0013	0.038	−0.00472	0.0012	0.000	−0.0077	0.0014	0.000
1Lx2L	−0.00107	0.0004	0.016	0.000762	0.0004	0.054	0.00161	0.0004	0.001

**Table 8:** Fit function parameters for the I_S=O,_ ΔI_S=O_ RTFOT, and ΔI_S=O_ RTFOT + PAV.

Effect	I_S=O_; R^2^ = 0.316; MSE = 0.0000001			ΔI_S=O_ RTFOT; R^2^ = 0.311; MSE = 0.0000003			ΔI_S=O_ RTFOT + PAV; R^2^ = 0.991; MSE = 0.0000013		
	RC	SE	*p* Value	RC	SE	*p* Value	RC	SE	*p* Value
Intercept	0.00189	0.00037	0.000	0.00110	0.00071	0.121	0.00473	0.00155	0.003
(1)SW_LC_ (L)	−0.00087	0.00043	0.049	0.00019	0.00082	0.817	0.00322	0.00180	0.078
SW_LC_ (Q)	0.00033	0.00012	0.007	−0.00001	0.00023	0.957	−0.0016	0.00050	0.002
(2) TOA (L)	0.00239	0.00064	0.000	−0.00137	0.00120	0.259	−0.0038	0.00265	0.160
TOA (Q)	−0.00270	0.00076	0.000	0.00253	0.00143	0.081	−0.0046	0.00315	0.150
1Lx2L	−0.00043	0.00024	0.078	−0.00083	0.00046	0.072	0.00422	0.00101	0.000

**Table 9:** Fit function parameters for the I_C=C,_ ΔI_C=C_ RTFOT, and ΔI_C=C_ RTFOT + PAV.

Effect	I_C=C_; R^2^ = 0.603; MSE = 0.0000004			ΔI_C=C_ RTFOT; R^2^ = 0.306; MSE = 0.0000015			ΔI_C=C_ RTFOT + PAV; R^2^ = 0.277; MSE = 0.0000003		
	RC	SE	*p* Value	RC	SE	*p* Value	RC	SE	*p* Value
Intercept	0.01241	0.00092	0.000	0.00367	0.00167	0.030	0.00302	0.00072	0.000
(1)SW_LC_(L)	−0.00013	0.00106	0.906	−0.0041	0.00193	0.039	−0.0015	0.00083	0.083
SW_LC_ (Q)	0.00031	0.00029	0.299	0.00121	0.00054	0.028	0.00029	0.00023	0.208
(2)TOA(L)	−0.00104	0.00156	0.509	0.00788	0.00284	0.007	−0.0006	0.00122	0.624
TOA (Q)	0.00427	0.00186	0.024	−0.0008	0.00338	0.818	−0.0008	0.00145	0.610
1Lx2L	−0.00263	0.00059	0.000	−0.0033	0.00108	0.003	0.00033	0.00046	0.484

**Table 10:** Parameters of the CAM model fitted to the experimental data—bitumen, bitumen + 1.5% SW_LC_, and bitumen + (1.5% SW_LC_ + 0.4% TOA).

Bitumen	Additives (%)		Aging Process	CAM Model Parameters							
	SW_LC_	TOA		Gg	k	m_e_	f_c_	C_1_	C_2_	R_c_	R^2^
50/70	0.0	0.0	Unaged	7.49 × 10^6^	0.095	1.571	11.555	10.7	138.9	3.17	0.974
	0.0	0.0	RTFOT	7.49∙× 10^6^	0.088	1.670	0.415	14.1	168.8	3.41	0.966
	0.0	0.0	RTFOT + PAV	7.49∙× 10^6^	0.079	1.938	0.001	15.6	175.0	3.83	0.971
	1.5	0.0	Unaged	7.49∙× 10^6^	0.092	1.468	11.332	11.7	14.5	3.26	0.945
	1.5	0.0	RTFOT	7.49∙× 10^6^	0.093	1.393	10.547	14.2	165.5	3.24	0.922
	1.5	0.0	RTFOT + PAV	7.49∙× 10^6^	0.080	1.797	0.004	16.4	178.0	3.77	0.928
	1.5	0.4	Unaged	7.49∙× 10^6^	0.090	1.443	11.480	11.9	140.7	3.34	0.964
	1.5	0.4	RTFOT	7.49∙× 10^6^	0.093	1.397	11.740	14.1	162.2	3.24	0.933
	1.5	0.4	RTFOT + PAV	7.49∙× 10^6^	0.081	1.817	0.006	15.3	167.9	3.72	0.949

## Data Availability

The original contributions presented in this study are included in the article. Further inquiries can be directed to the corresponding author.

## Abbreviations

The following abbreviations are used in this manuscript:

SW_LC_	Synthetic wax low-carbon
TOA	Tall oil amidoplyamine
DSR	Dynamic shear rheometer
MSCR	Multiple stress creep recovery test
BBR	Bending beam rheometer
FTIR	Fourier transform infrared spectroscopy
RTFOT	Rolling thin film oven test
PAV	Pressure aging vessel
PRR	Penetration retention ratio
SPI	Softening temperature increment
VAI	Viscosity aging index
